# Immunomodulatory Effects of Pneumococcal Extracellular Vesicles on Cellular and Humoral Host Defenses

**DOI:** 10.1128/mBio.00559-18

**Published:** 2018-04-10

**Authors:** Mario Codemo, Sandra Muschiol, Federico Iovino, Priyanka Nannapaneni, Laura Plant, Sun Nyunt Wai, Birgitta Henriques-Normark

**Affiliations:** a Department of Microbiology, Tumor and Cell Biology, Karolinska Institutet and Department of Clinical Microbiology, Karolinska University Hospital, Stockholm, Sweden; b Department of Molecular Biology and the Umeå Centre for Microbial Research (UCMR), Umeå University, Umeå, Sweden; c Singapore Centre on Environmental Life Sciences Engineering and Lee Kong Chian School of Medicine (LKC), Nanyang Technological University (NTU), Singapore, Singapore; GSK Vaccines

**Keywords:** *Streptococcus pneumoniae*, cellular and humoral defense, complement, extracellular vesicles, pneumococcal evasion of immune response, pneumococci, pneumolysin

## Abstract

Gram-positive bacteria, including the major respiratory pathogen Streptococcus pneumoniae, were recently shown to produce extracellular vesicles (EVs) that likely originate from the plasma membrane and are released into the extracellular environment. EVs may function as cargo for many bacterial proteins, however, their involvement in cellular processes and their interactions with the innate immune system are poorly understood. Here, EVs from pneumococci were characterized and their immunomodulatory effects investigated. Pneumococcal EVs were protruding from the bacterial surface and released into the medium as 25 to 250 nm lipid stained vesicles containing a large number of cytosolic, membrane, and surface-associated proteins. The cytosolic pore-forming toxin pneumolysin was significantly enriched in EVs compared to a total bacterial lysate but was not required for EV formation. Pneumococcal EVs were internalized into A549 lung epithelial cells and human monocyte-derived dendritic cells and induced proinflammatory cytokine responses irrespective of pneumolysin content. EVs from encapsulated pneumococci were recognized by serum proteins, resulting in C3b deposition and formation of C5b-9 membrane attack complexes as well as factor H recruitment, depending on the presence of the choline binding protein PspC. Addition of EVs to human serum decreased opsonophagocytic killing of encapsulated pneumococci. Our data suggest that EVs may act in an immunomodulatory manner by allowing delivery of vesicle-associated proteins and other macromolecules into host cells. In addition, EVs expose targets for complement factors in serum, promoting pneumococcal evasion of humoral host defense.

## IMPORTANCE

Streptococcus pneumoniae is a major contributor to morbidity and mortality worldwide, being the major cause of milder respiratory tract infections such as otitis and sinusitis and of severe infections such as community-acquired pneumonia, with or without septicemia, and meningitis. More knowledge is needed on how pneumococci interact with the host, deliver virulence factors, and activate immune defenses. Here we show that pneumococci form extracellular vesicles that emanate from the plasma membrane and contain virulence properties, including enrichment of pneumolysin. We found that pneumococcal vesicles can be internalized into epithelial and dendritic cells and bind complement proteins, thereby promoting pneumococcal evasion of complement-mediated opsonophagocytosis. They also induce pneumolysin-independent proinflammatory responses. We suggest that these vesicles can function as a mechanism for delivery of pneumococcal proteins and other immunomodulatory components into host cells and help pneumococci to avoid complement deposition and phagocytosis-mediated killing, thereby possibly contributing to the symptoms found in pneumococcal infections.

## INTRODUCTION

Streptococcus pneumoniae (the pneumococcus) is responsible for a substantial morbidity and mortality worldwide. About 1 million children below 5 years of age die due to pneumococcal infections every year globally (1). Pneumococci are major causes of community-acquired pneumonia, septicemia, and meningitis but are also the main contributor to less severe respiratory infections such as otitis media and sinusitis.

All cell types can form extracellular vesicles (EVs) by membrane budding and outward pinching off of spherical membrane particles. In Gram-negative bacteria, EVs may be formed by budding from the outer membrane, forming so-called outer membrane vesicles (OMVs) (2). These OMVs range in size from 10 to 300 nm and contain components of the outer membrane as well as acting as a cargo primarily derived from the periplasmic space. OMVs have been shown to have many functions such as effects on bacterial virulence but have also been suggested to act as a mechanism for delivery of virulence factors to host cells, as well as to act a decoy for immune evasion by bacteria (3–5). Only recently, membrane-derived EVs were discovered in Gram-positive bacteria that lack an outer membrane and where the cytoplasmic membrane is covered by a thick peptidoglycan cell wall (3, 6). The mechanisms resulting in plasma membrane-derived EVs are not known, but the different origins of OMVs from Gram-negative bacteria and of EVs from Gram-positive bacteria result in different cargos of proteins and other macromolecules. In Staphylococcus aureus, EVs have been shown to deliver α-hemolysin to host cells, causing cytotoxicity (7, 8). EVs from Bacillus anthracis have been characterized using proteomic approaches, and a biologically active toxin was found in those EVs (9). Recently, it was shown that pneumococci also produce EVs (10). Proteomic analysis of EVs from the nonencapsulated strain R6 showed differential enrichment of proteins localized to the plasma membrane fraction compared to a total bacterial lysate. Furthermore, EVs from a serotype 8 strain were shown to be protective in BALB/c mice against homologous challenge, but the nature of protection was not defined (10).

Here we characterize EVs from the pneumococcal strain TIGR4 (T4) of serotype 4 and examine the immunomodulatory effects of EVs from wild-type and pneumolysin-deficient pneumococci on epithelial and immune cells. We provide evidence that pneumococcal EVs are enriched for the active form of the major cytotoxin pneumolysin, suggesting that release of this cytosolic protein may occur through plasma membrane budding and that EVs may deliver pneumolysin into host cells. We further demonstrate that EVs, in contrast to encapsulated pneumococci, avidly bind components of the complement system, thereby depleting human serum of complement factor C3 and decreasing pneumococcal opsonophagocytosis.

## RESULTS

### Pneumococcal extracellular vesicles (EVs) contain several pneumococcal surface proteins and are enriched for the cytotoxin pneumolysin.

**FIG 1:**
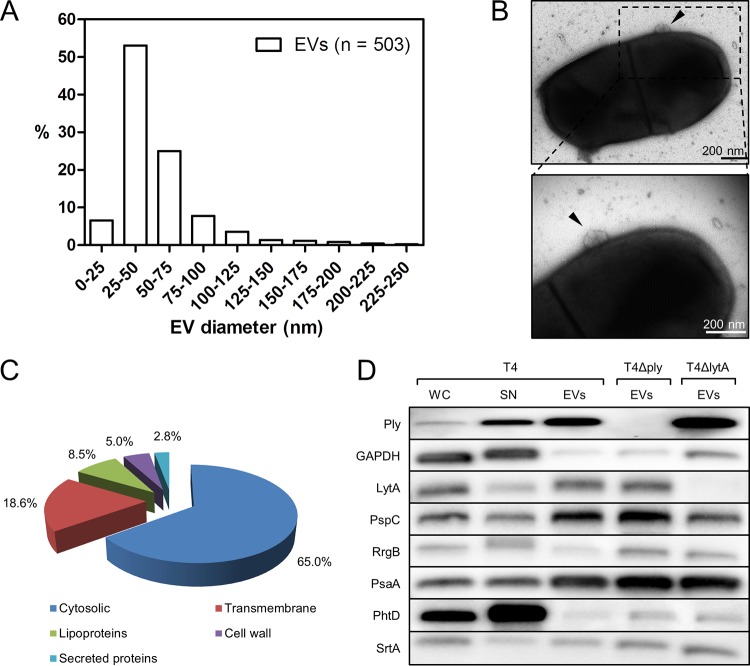
Characterization of extracellular vesicles (EVs) produced by pneumococcal strain T4 (TIGR4). (A) Size distribution of pneumococcal EVs from strain T4. (B) Electron micrograph of vesicles budding from S. pneumoniae strain T4R (top) and the same vesicles in higher magnification (bottom). The arrowheads point at released EVs. (C) Mass spectrometry identification of pneumococcal proteins present in EVs. Numbers indicate percentages of identified proteins, defined as cytosolic proteins, membrane-associated proteins (lipoproteins), transmembrane proteins, cell wall-associated proteins, and secreted proteins, based on their subcellular localization. (D) Immunoblot detection of pneumococcal proteins and virulence factors present in EVs isolated from pneumococcal strain T4 and from its isogenic mutants deficient in pneumolysin (T4Δ*ply*) or LytA (T4Δ*lytA*). In comparison, proteins present in the whole-cell lysate (WC) and supernatant (SN) of a liquid bacterial culture are visualized.

We isolated and purified extracellular vesicle fractions (EVs) from S. pneumoniae serotype 4 strain T4 (TIGR4) grown in liquid C + Y medium (Casitone [5.0 g], yeast extract [1.0 g]). Electron and atomic force microscopy revealed spherical EVs surrounded by membranous structures (see Fig. S1A and B in the supplemental material). The released EVs showed diameters ranging from less than 25 nm to 250 nm (Fig. 1A). To reveal budding of EVs from the surface of bacteria, we used isogenic capsular-deficient mutant T4R to avoid interference by the thick bacterial capsule. EVs were observed as spherical vesicles on the surface of bacterial cells by transmission electron microscopy. For most bacteria examined, few EVs were usually found per bacterium that could emerge from the entire surface, including the cell pole (Fig. 1B; see also Fig. S1C).

10.1128/mBio.00559-18.1 FIG S1  Visualization of EVs and identification of proteins present in EV preparation. (A) Electron micrograph of EVs after isolation and density gradient purification from S. pneumoniae strain T4 (TIGR4). (B) Atomic force micrograph of pneumococcal EVs (left) and of the same sample in three dimensions (3D) (right). (C) Electron micrographs of the isogenic nonencapsulated mutant of T4 (T4R), with released EVs indicated by black arrows. (D) An EV preparation was separated by SDS-PAGE, and proteins were revealed by Coomassie staining (right lane; EVs). In comparison, proteins present in the whole-cell lysate (WC) and culture supernatant (SN) of a liquid bacterial culture were visualized. Download FIG S1, TIF file, 1.2 MB. Copyright © 2018 Codemo et al. 2018 Codemo et al. This content is distributed under the terms of the Creative Commons Attribution 4.0 International license.

SDS-PAGE performed on EV preparations from strain T4 revealed a protein pattern diverging from the patterns exhibited by a total bacterial extract and the corresponding supernatant (see Fig. S1D). Proteins in EV preparation were identified by tandem mass spectrometry, and their subcellular localization was predicted. Most of them appeared to be proteins present in the cytosol (206 of 317 proteins [65%]). A smaller percentage were proteins with at least one transmembrane domain (59/317 [18.6%]), lipoproteins (27/317 [8.5%]), proteins anchored to the cell wall with choline-binding domain or LPxTG anchor (16/317 [5%]), or secreted proteins (9/317 [2.8%]) (Fig. 1C; see also Table S1 in the supplemental material).

10.1128/mBio.00559-18.8 TABLE S1  Mass spectrometry identification of pneumococcal proteins present in EVs and their topology prediction. Download TABLE S1, DOCX file, 0.1 MB. Copyright © 2018 Codemo et al. 2018 Codemo et al. This content is distributed under the terms of the Creative Commons Attribution 4.0 International license.

EVs were shown to contain only some of the major known surface-associated pneumococcal virulence proteins and appeared to be enriched for proteins containing choline-binding domains (known to bind to choline residues on membrane-associated lipoteichoic acids) (see Table S2). In contrast, only a few of the peptidoglycan-anchored LPxTG proteins were identified in the EVs by mass spectrometry, suggesting that the EVs were budding through the peptidoglycan layer and did not contain much cell wall material. Known plasma membrane proteins such as the sialic acid ABC transporter SatA and pyruvate oxidase SpxB were identified. Both pneumolysin and the LytA amidase, lacking SecA targeting signal sequences, were present in EVs. Immunoblot analyses showed enrichment for some proteins with respect to the culture supernatant from which EVs were isolated and from a total bacterial lysate (Fig. 1D). Pneumolysin in particular was enriched in EVs, in contrast to another cytosolic protein, GAPDH (glyceraldehyde-3-phosphate dehydrogenase), that can be released by pneumococci. Enrichment of pneumolysin was also seen in EVs isolated from a *lytA* mutant (T4Δ*lytA*) (11) deficient in autolysis, suggesting that pneumolysin is localized within EVs as part of a cytosolic cargo. Pneumolysin expression was not required for EV formation, as EVs could also be isolated from a pneumolysin-deficient mutant (T4Δ*ply*) (12) with similar protein profiles (Fig. 1D). Both the LytA and PspC choline-binding proteins were enriched in EVs from T4, which was not the case for the LPxTG major pilin subunit RrgB (13–15). Interestingly, pneumococcal surface antigen A (PsaA), a manganese binding surface protein in S. pneumoniae that is docked to a membrane-bound ABC manganese transporter, was highly enriched in EVs (16). This is in contrast to the pneumococcal histidine triad protein PhtD, a zinc-scavenging surface protein that was released into the medium and that is not known to be firmly docked to membrane transporters (17). Thus, our data suggest that pneumococcal EVs are derived from the plasma membrane and contain a cytoplasmic protein cargo as well as surface proteins docked to the outer surface of the vesicles.

10.1128/mBio.00559-18.9 TABLE S2  Pneumococcal virulence factors and their presence in EVs. Download TABLE S2, DOCX file, 0.02 MB. Copyright © 2018 Codemo et al. 2018 Codemo et al. This content is distributed under the terms of the Creative Commons Attribution 4.0 International license.

### EVs are internalized by human lung epithelial cells, allowing delivery of pneumolysin into host cells.

**FIG 2:**
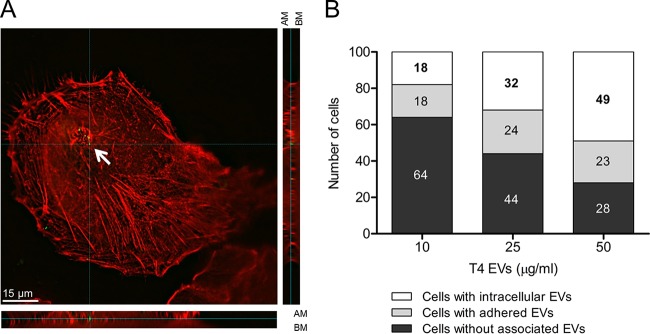
EVs are internalized by A549 lung epithelial cells. (A) High-resolution immunofluorescence microscopy imaging of A549 cells incubated with EVs (50 μg/ml) for 6 h and stained with phalloidin, used as an epithelial cell marker (red), and pneumolysin, used as an EV marker (green). In this representative image, the complete thickness of epithelial cells has been imaged in 21 z-stacks. The internalized vesicle, indicated with a white arrow, has been captured in z-stack 11, which is displayed from the top (A) and in *XZ* (A [bottom]) in *YZ* (A [right]) orthogonal views to demonstrate intracellular localization. AM, apical membrane; BM, basolateral membrane. (B) Quantification of internalization of vesicles in A549 cells treated with 10, 25, and 50 µg/ml of EVs from T4. For each concentration, 100 cells were analyzed and the number of cells with intracellular or extracellular signal or of cells without EV-associated signal is shown.

To gain insight into the effects of pneumococcal EVs on host cells, we incubated A549 lung epithelial cells with EVs from strain T4 or its isogenic mutant deficient in pneumolysin (T4Δ*ply*) for 24 h. No significant differences with respect to cell death were detected in samples incubated with EVs or left untreated (see Fig. S2). Purified pneumolysin added to the cell medium at concentrations similar to those present in EVs (see Fig. S3) did not display toxicity toward A549 cells, suggesting that the lack of toxicity of EVs may not be due to a biologically inactive pneumolysin. Next, we asked if EVs can be internalized by epithelial cells and analyzed immunofluorescence micrographs of A549 cells treated with increasing concentrations of T4 EVs (see Fig. S4A). Orthogonal views of EV-treated A549 cells enabled us to view the cell monolayer from the *x*, *y* and *z* axes (Fig. 2A). Pneumolysin-containing EVs were observed inside the red layer of A549 cells corresponding to actin staining, in both the *XZ* and *YZ* perspectives, indicating intracellular localization of EVs. When we quantified the number of cells with intracellular EVs after incubation with 10, 25, or 50 µg/ml of EVs from T4, a dose-dependent increase in the number of cells with internalized EVs was observed (Fig. 2B).

10.1128/mBio.00559-18.2 FIG S2  Viability of A549 cells after treatment with EVs and purified pneumolysin. Viability of A549 cells was examined by flow cytometry of fixable viability dye (FVD)-positive cells after 24 h of incubation with different concentrations of EVs (10, 25, and 50 μg/ml) from the wild-type T4 strain or its isogenic mutant deficient in pneumolysin (T4Δ*ply*) or purified pneumolysin (0.55, 1.375, or 2.75 μg/ml). As a control treatment, A549 cells were incubated with PBS (−) or with detergent NP-40 (0.02%)-PBS for 30 min. Data are represented as means ± SEM of results from three independent experiments. Download FIG S2, TIF file, 0.2 MB. Copyright © 2018 Codemo et al. 2018 Codemo et al. This content is distributed under the terms of the Creative Commons Attribution 4.0 International license.

10.1128/mBio.00559-18.3 FIG S3  Quantification of pneumolysin in EVs. (A) Quantification of pneumolysin present in EVs by immunoblotting. One microgram of EVs (in terms of the total protein quantity) or of purified pneumolysin was separated by SDS-PAGE and analyzed by immunoblotting. Dotted lines indicate where lanes from the same blot were spliced together. (B) The quantity of pneumolysin present in EVs was calculated from three independent Western blot images with respect to purified pneumolysin. Data are represented as means ± SEM of results from three independent experiments. Download FIG S3, TIF file, 0.2 MB. Copyright © 2018 Codemo et al. 2018 Codemo et al. This content is distributed under the terms of the Creative Commons Attribution 4.0 International license.

10.1128/mBio.00559-18.4 FIG S4  Immunofluorescence microscopy imaging of A549 cells and DCs treated with EVs from strain T4. (A) A549 cells were incubated with increasing concentrations of EVs (10, 25, and 50 μg/ml) for 6 h and stained with phalloidin (red) and pneumolysin, which was used as an EV marker (green). Bar, 25 µm. (B) DCs were incubated with increasing concentrations of EVs (10, 25, and 50 μg/ml) for 2 h and stained for actin (red) and pneumolysin, which was used as an EV marker (green). Bar, 25 µm. Download FIG S4, TIF file, 1 MB. Copyright © 2018 Codemo et al. 2018 Codemo et al. This content is distributed under the terms of the Creative Commons Attribution 4.0 International license.

### EVs activate human monocyte-derived dendritic cells (DCs) and induce proinflammatory cytokine responses.

**FIG 3:**
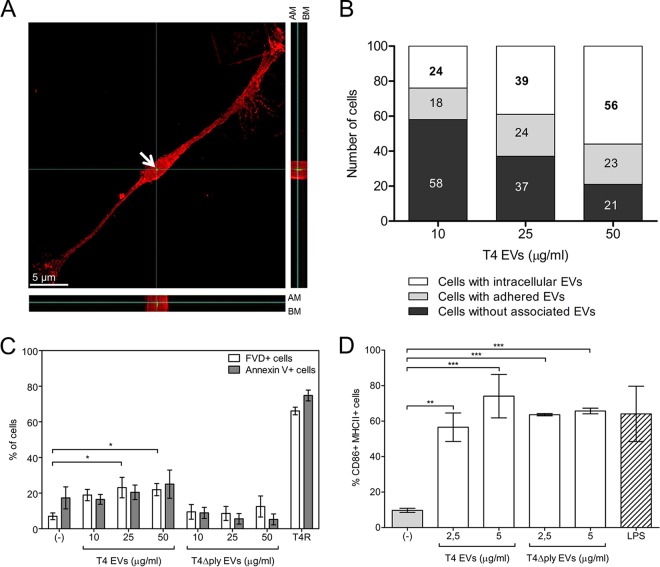
EVs are internalized by and activate human monocyte-derived dendritic cells (DCs). (A) High-resolution immunofluorescence microscopy imaging of DCs incubated with EVs (50 μg/ml) for 2 h and stained with phalloidin, used as a DC marker (red), and pneumolysin, used as an EV marker (green). In this representative image, the complete thickness of DCs has been imaged in 21 z-stacks; the internalized vesicle, indicated with a white arrow, has been captured in z-stack 10, which is displayed from the top (A) and in *XZ* (A [bottom]) and in *YZ* (A [right]) orthogonal views to show the intracellular localization of the vesicles. AM, apical membrane; BM, basolateral membrane. (B) Quantification of internalization of vesicles in DCs treated with 10, 25, and 50 µg/ml of EVs from T4. For each concentration, 100 cells were analyzed and the number of cells with intracellular or extracellular signal or of cells without EV-associated signal is shown. (C) Percentages of fixable viability dye (FVD)- and annexin V-positive cells analyzed by flow cytometry. Cells were incubated for 24 h with different concentrations (10, 25, and 50 μg/ml) of EVs isolated from the wild-type T4 strain or its isogenic mutant deficient in pneumolysin (T4Δ*ply*) or, as a control in the capsule, the nonencapsulated strain T4R or PBS (−). (D) DC activation measured by flow cytometry of MHC-II- and CD86-positive cells after 24 h of incubation with different concentrations (2.5 and 5 μg/ml) of EVs from strain T4 or mutant T4Δ*ply*, LPS (1 μg/ml), or PBS (−). Data are represented as means ± standard errors of the means (SEM) of results from three independent experiments. *, *P* < 0.05; **, *P* < 0.01; ***, *P* < 0.001.

Along with epithelial cells, DCs play an important role in pneumococcal infection. We therefore investigated uptake of EVs from strain T4 by DCs using immunofluorescence microscopy (see Fig. S4B). Orthogonal views of DCs incubated with EVs showed pneumolysin-containing EVs inside the cells (Fig. 3A). A dose-dependent increase in the number of DCs with intracellular EVs was observed (Fig. 3B). To assess whether EVs have cytotoxic effects on DCs, DCs were incubated with EVs from T4 and stained with fixable viability dye and annexin V followed by flow cytometry. EVs isolated from wild-type bacteria were associated with a slight increase in cell death rates, while EVs from the pneumolysin-deficient mutant T4Δ*ply* failed to induce cell death, suggesting that the toxicity of EVs in DCs is pneumolysin dependent. However, pneumolysin-containing EVs were considerably less cytotoxic than live bacteria (Fig. 3C). The percentage of annexin V-positive cells was lower in cells treated with EVs isolated from the T4Δ*ply* strain than in untreated cells. This difference was, however, not statistically significant and could have been a result of biological variation between different donors.

**FIG 4:**
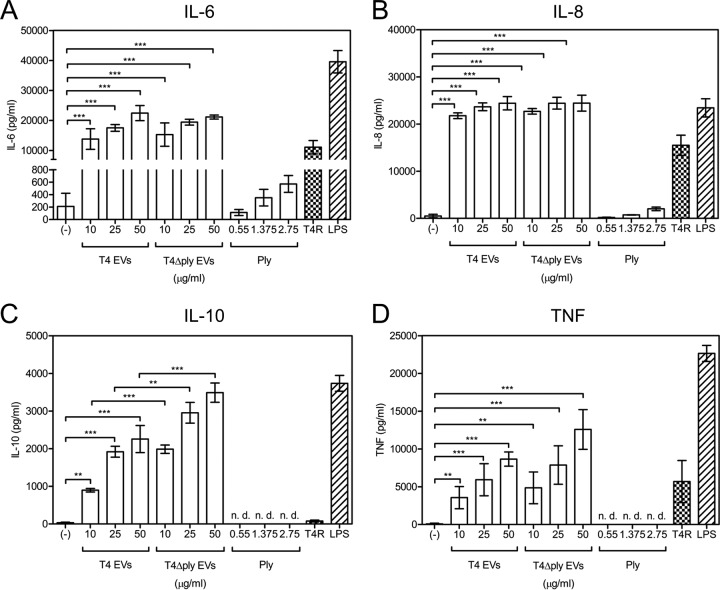
EVs induce cytokine release by human monocyte-derived dendritic cells. Induction of (A) IL-6, (B) IL-8, (C) IL-10, and (D) TNF by DCs after a 24-h incubation with different concentrations (10, 25, and 50 μg/ml) of EVs from strain T4 or mutant T4Δ*ply* or purified pneumolysin (0.55, 1.375, or 2.75 μg/ml). Control treatments included PBS (−), the nonencapsulated isogenic mutant of T4 (T4R), and LPS (1 μg/ml). n.d., not detectable. Data are represented as means ± SEM of results from three independent experiments. **, *P* < 0.01; ***, *P* < 0.001.

Presentation of processed antigens by activated DCs is achieved after expression of major histocompatibility complex class II (MHC-II) and costimulatory molecules on the cell surface. To assess if uptake of EVs resulted in activation of DCs, cells were incubated with EVs from T4, stained for MHC-II and the CD86 costimulatory molecule, and analyzed by flow cytometry. A dose-dependent increase in the levels of the two surface molecules was detected when DCs were incubated with EVs from T4 (Fig. 3D). Moreover, EVs from the pneumolysin-deficient strain T4Δ*ply* induced activation of DCs to the same extent as EVs from T4, suggesting that DC activation is pneumolysin independent. We also determined the consequences of DC activation in terms of cytokine responses. Culture supernatants were analyzed using enzyme-linked immunosorbent assays (ELISAs) to determine concentrations of interleukin-6 (IL-6) (Fig. 4A), IL-8 (Fig. 4B), IL-10 (Fig. 4C), and tumor necrosis factor (TNF) (Fig. 4D). The EVs induced expression of all four cytokines to higher levels than were seen with live bacteria but failed to induce release of IL-1β or IL-12 into the supernatant (see Fig. S5). Incubation of DCs with EVs isolated from the T4Δ*ply* mutant also resulted in increased production of proinflammatory cytokines and an even higher level of secretion of the anti-inflammatory cytokine IL-10 (Fig. 4C). Stimulation with purified pneumolysin, at concentrations similar to those found in EVs (see Fig. S3), induced secretion of only IL-6 and IL-8 but at very low levels compared to what was found in supernatants from DCs stimulated with EVs. Overall, these data suggest that the cytokine release induced by pneumococcal EVs is pneumolysin independent.

10.1128/mBio.00559-18.5 FIG S5  Release of IL-1β and IL-12 p70 by DCs. Cells were incubated for 24 h with different concentrations of EVs (10, 25, and 50 μg/ml), and induction of (A) IL-1β and (B) IL-12 p70 was measured by ELISA. As control treatments, DCs were incubated with PBS (−), the nonencapsulated isogenic mutant of strain T4 (T4R), or LPS (1 μg/ml). Data are represented as means ± SEM of results from three independent experiments. Statistical analysis was performed by one-way ANOVA test and Dunnett’s posttest. n.s., not significant. Download FIG S5, TIF file, 0.3 MB. Copyright © 2018 Codemo et al. 2018 Codemo et al. This content is distributed under the terms of the Creative Commons Attribution 4.0 International license.

### EVs are lipid-containing vesicles harboring the factor H recruiting protein PspC.

**FIG 5:**
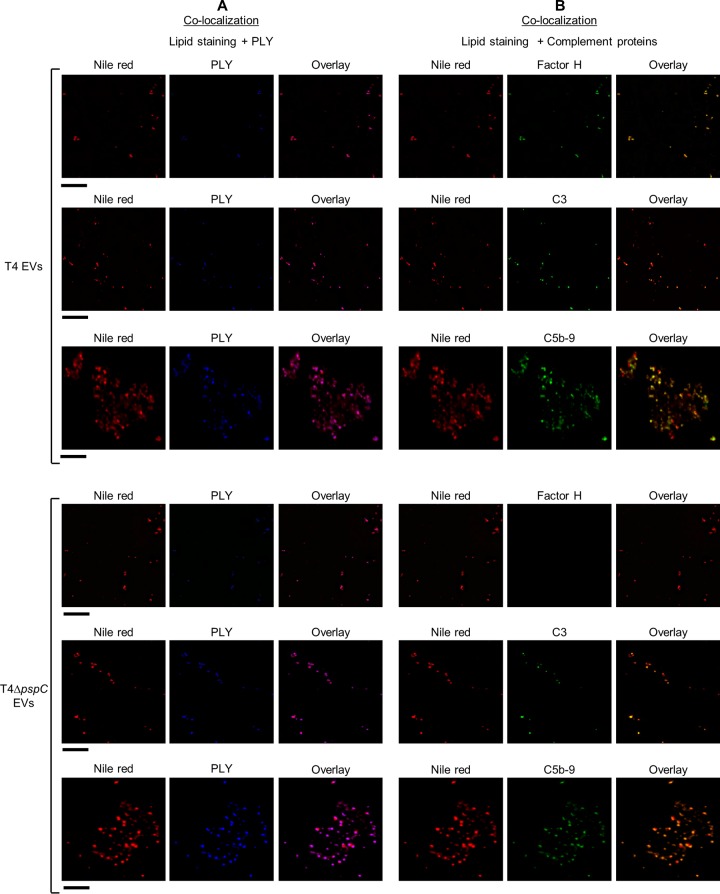
EVs are lipid-containing vesicles that bind human complement proteins. Results of high-resolution immunofluorescence microscopy of EVs from strain T4 or its isogenic mutant deficient in the factor H binding protein PspC (T4Δ*pspC*) after incubation with human serum are shown. (A) Colocalization of lipid staining using Nile red (red) and pneumolysin (PLY) (blue). Colocalization of PLY with Nile red confirmed that Nile red stains pneumococcal EVs. (B) Colocalization of lipid staining using Nile red (red) and complement proteins factor H, C3, and C5b-9 (green). Black bars represent 5 µm.

To verify that pneumococcal EVs are lipid-containing vesicles, we first performed high-resolution immunofluorescence microscopy examination of EVs using the lipid stain Nile red and showed colocalization of EVs stained with Nile red and pneumolysin (Fig. 5A). To investigate if the EVs were orientated inside out or, as expected, outside out, we made use of the main factor H (FH)-recruiting protein in pneumococci, PspC. In intact bacteria, PspC is surface bound to choline residues on plasma membrane-anchored lipoteichoic acids. When we incubated EVs from T4 with human serum, we noted avid FH binding to the Nile red-stained EVs. No occurrence of FH recruitment to EVs isolated from a *pspC* mutant of T4 (T4Δ*pspC*) was seen (Fig. 5B). This suggests that pneumococcal EVs expose PspC on their surface, suggesting that budding EVs retain the same outside-out orientation as the plasma membrane.

### EVs expose targets for complement C3b deposition and formation of membrane attack complexes.

**FIG 6:**
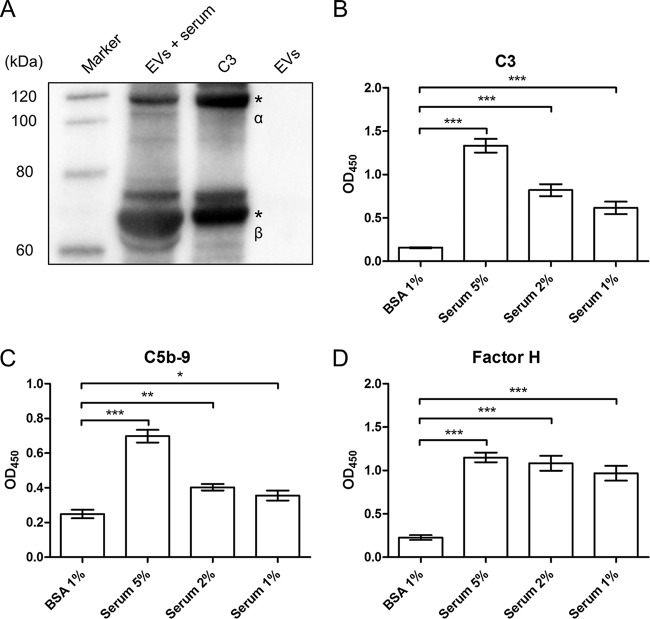
EVs recruit complement proteins such as human C3, factor H, and C5b-9. (A) Immunoblot detection of human C3 binding to EVs (α′-chain, 110 kDa; β-chain, 75 kDa). Lane 1, EVs after incubation with human serum for 2 h at 37°C; lane 2, purified human C3 (1 μg); lane 3, purified EVs (1 μg). (B to D) Detection of (B) C3, (C) C5b-9, and (D) factor H (FH) binding to EVs using ELISAs. Data are represented as means ± SEM of results from three independent experiments. *, *P* < 0.05; **, *P* < 0.01; ***, *P* < 0.001.

In encapsulated pneumococci, the capsule acts as an efficient barrier against deposition of complement factor C3b, restricting complement deposition to underlying targets. C3b binding, which initiates the alternative pathway of the complement system, leads to the formation of pore-forming membrane attack complexes (MAC) by formation of membrane-spanning terminal complement complex C5b-9 (18). To assess if EVs can deposit C3b or C5b-9 complexes, we incubated EVs from T4 with human serum and performed immunofluorescence microscopy (Fig. 5B). Depositions of C3b and C5b-9 complexes occurred on virtually all Nile red-stained EVs. The staining efficiency for C3b and C5-9 was not affected by the presence or absence of the FH-recruiting protein PspC. To further show the binding of C3b to EVs, we performed Western blotting and ELISAs on EVs incubated with human serum. While Western blotting confirmed the binding of C3 to EVs (Fig. 6A), ELISAs also showed increased levels of binding with increasing concentrations of the serum used (Fig. 6B). The same result was observed in ELISAs for C5b-9 (Fig. 6C) and FH (Fig. 6D) binding to EVs. In addition, we performed SDS-PAGE on EVs after incubation with human serum (see Fig. S6) and performed mass spectrometry on selected bands (see Table S3). Proteins from both the classical and alternative pathways of the complement system were found to bind to EVs, as did apolipoprotein B-100, which is known to bind pneumolysin (19).

10.1128/mBio.00559-18.6 FIG S6  Binding of human serum proteins to EVs. EVs (50 μg/ml) were incubated with human serum (20% final concentration) for 2 h at 37°C and separated by SDS-PAGE. Proteins were visualized by Coomassie staining. As a reference, purified EVs (lane 1) and human serum (lane 3) were loaded. Bands subjected to mass spectrometry analysis are indicated by arrows. Download FIG S6, TIF file, 0.6 MB. Copyright © 2018 Codemo et al. 2018 Codemo et al. This content is distributed under the terms of the Creative Commons Attribution 4.0 International license.

10.1128/mBio.00559-18.10 TABLE S3  Human serum proteins bound by pneumococcal EVs. Download TABLE S3, DOCX file, 0.01 MB. Copyright © 2018 Codemo et al. 2018 Codemo et al. This content is distributed under the terms of the Creative Commons Attribution 4.0 International license.

### Addition of pneumococcal EVs to human serum impairs the phagocytic activity of macrophages.

**FIG 7:**
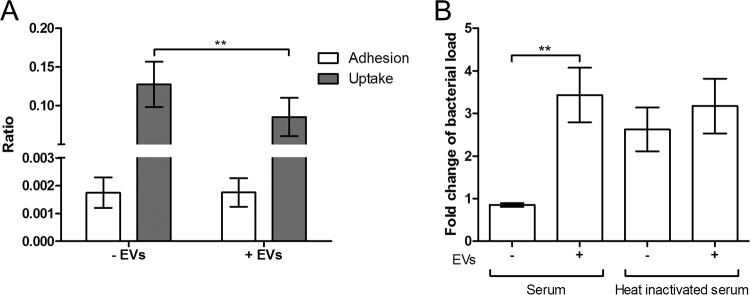
EVs bind complement proteins in human serum, thereby impairing the phagocytic activity of host cells. (A) Adhesion and phagocytosis assay of THP-1 cells incubated with pneumococcal strain T4 pretreated with human serum only (-EVs) or with human serum and EVs (+ EVs) for 30 min at 37°C. (B) Opsonophagocytosis killing assay of differentiated HL-60 cells incubated with strain T4 pretreated with human serum or heat-inactivated human serum, alone or in combination with EVs. Data are represented as means ± SEM of results from three independent experiments. *, *P* < 0.05; **, *P* < 0.01.

To assess whether sequestration of complement proteins by EVs would reduce phagocytosis of bacteria, we first incubated human THP-1 macrophages with T4 bacteria preincubated with human serum alone or with human serum incubated with EVs. Addition of EVs did not influence bacterial adhesion to the cells, but bacteria were internalized less efficiently by the macrophages (Fig. 7A). To determine if the presence of EVs in the sera would affect the overall survival of bacteria in an opsonophagocytosis-killing assay, we incubated T4 bacteria with differentiated HL-60 human granulocytes after opsonization with human serum. While granulocytes were able to kill bacteria treated with human serum at moderate rates, sequestration of complement components by EVs led to inhibition of bacterial killing by granulocytes to the same extent as incubating bacteria with heat-inactivated human serum (Fig. 7B).

## DISCUSSION

Extracellular vesicles (EVs) have been shown to be formed by a number of bacteria and have been found to contain virulence factors that can be delivered to host cells during the infection process (9). EVs have been extensively studied and characterized in Gram-negative bacteria but less extensively in Gram-positive bacteria. Since Gram-positive bacteria lack an outer membrane, EVs are expected to be enriched for macromolecules associated with the plasma membrane and to contain a cargo of cytosolic constituents. Here we characterized EVs produced by the encapsulated pneumococcal strain TIGR4 (T4) and studied their immunomodulatory effects. We found that pneumococcal EVs contain plasma membrane constituents such as lipids and membrane proteins as well as choline-binding surface proteins associated with the plasma membrane via choline residues on lipoteichoic acids. Also, known proteins docked to the membrane by forming complexes to ATP transporters, such as the manganese protein PsaA, were enriched in EV preparations. Cell wall-anchored LPxTG proteins were not enriched, suggesting a low content of cross-linked peptidoglycan.

In a previous study by Olaya-Abril et al. (10), extracellular vesicles were isolated from five pneumococcal strains belonging to serotypes 23F, 6B, 8, 1, and from the nonencapsulated strain R6. Vesicles were prepared from bacteria grown in Todd-Hewitt broth medium until the late exponential phase (optical density [OD], 0.5), corresponding to growth conditions that were similar overall to those we have used in our study (C + Y medium; OD, 0.9). In line with the study by Olaya-Abril et al., most pneumococcal vesicles isolated from the T4 strain had similar sizes ranging from 20 to 80 nm, but a small proportion of T4 EVs were up to 250 nm in size. The total number of proteins identified by mass spectrometry in EVs from serotypes 23F, 6B, 8, 1, and R6 differed greatly and spanned a range from 181 proteins (EVs from serotype 8) to 373 proteins (EVs from serotype 6B). In our study, we identified a total of 317 proteins, the majority being cytoplasmic proteins and lipoproteins, similarly to what has been described previously (10). Interestingly, there was a marked variation of proteins present in EVs from serotypes 23F, 6B, 8, 1, and R6, suggesting serotype-specific protein content. Among the 10 core proteins that were found to be common in EVs from ≥4 strains, we identified 9 proteins (PspA, ABC-SBP [SP_0148], SatA, PBP1B, AmiA, MalX, PnrA, PrsA, and LytC) in EVs from strain T4. With regard to pneumococcal virulence factors, T4 vesicles were enriched in the pore-forming toxin pneumolysin and the autolysin LytA. LytA was not identified by mass spectrometry in EVs from serotypes 23F, 6B, 8, 1, and R6, and pneumolysin was identified in EVs from only two (23F and 6B). However, pneumolysin was detected in all EVs by immunogold staining and electron microscopy (10). Our data suggest that pneumolysin and LytA are not required for EV formation and that EVs may be formed from the plasma membrane such that cytosolic proteins associated with the inner leaflet of the membrane may be preferentially included in the vesicle cargo. If that was the case, the major portion of pneumolysin in EVs would be expected to be inside the vesicles. This is supported by our finding that the pneumolysin in the EVs had lower hemolytic activity than similar amounts of purified pneumolysin (see Fig. S7 in the supplemental material).

10.1128/mBio.00559-18.7 FIG S7  Hemolytic activity of EVs. Human blood from buffy coats was incubated for 1 h at 37°C with different concentrations of EVs from strain T4 or mutant T4Δ*ply* (1, 8, 20, 60, and 100 μg/ml) or purified pneumolysin (0.055, 0.44, 1.1, 3.3, and 5.5 μg/ml). As a control treatment, blood was incubated with PBS (−) or with 0.1% Triton X-100–PBS for 10 min (+). Data are represented as means ± SEM of results from three independent experiments. **, *P* < 0.01; ****, *P* < 0.0001. Download FIG S7, TIF file, 0.2 MB. Copyright © 2018 Codemo et al. 2018 Codemo et al. This content is distributed under the terms of the Creative Commons Attribution 4.0 International license.

The outer leaflet of the plasma membrane in pneumococci exposes membrane-anchored lipoteichoic acids that carry choline residues binding the pneumococcal set of choline-binding proteins. Choline-binding protein PspC is the main factor H (FH)-recruiting protein in pneumococci, protecting the organism against activation of the alternative pathway of the complement system (14, 15). Pneumococcal EVs efficiently recruit FH from human serum in a PspC-dependent fashion, suggesting that PspC is directly exposed to serum components on the outer side of the vesicles.

We next investigated potential interactions of pneumococcal EVs with epithelial cells and DCs. Using high-resolution fluorescence microscopy analysis, we observed intracellular localization of pneumolysin, using phalloidin to visualize actin filaments, and hence conclude that EVs are internalized by A549 lung epithelial cells. Also, no cytotoxic effects of pneumolysin in EVs were found on these cells. We suggest that uptake of EVs into host cells might be a mechanism for pneumococci to deliver pneumolysin and other immunomodulatory EV content into host cells.

EVs were also efficiently phagocytosed by primary human DCs, and a modest pneumolysin-dependent cell death rate was observed compared to the potent cytotoxic effect mediated by intact T4R bacteria. It is known that different cell types have different levels of sensitivity to pneumolysin (20). This difference in the levels of sensitivity to cholesterol-dependent cytolysins could be due to differences in the content and structure of cholesterol in the plasma membrane (21) but also by which mechanisms EVs become internalized compared to intact bacteria. It has been shown that OMVs from Salmonella enterica serovar Typhimurium are potent activators and immunomodulators of DCs (22). We found here that incubation of DCs with pneumococcal EVs resulted in expression of the MHC-II surface marker and the CD86 costimulatory molecule, which is a clear indication of dendritic cell maturation. EVs stimulated production of IL-6, IL-8, IL-10, and TNF but failed to induce the release of cytokines IL-1β and IL-12p70. It has previously been shown that DCs produce IL-10 when stimulated with lipopolysaccharide (LPS) in order to modulate their mature state after LPS stimulation (23). In agreement with these data, we suggest that the same mechanism applies to DCs incubated with pneumococcal EVs. Interestingly, EVs from a pneumolysin-deficient mutant induced DC activation and cytokine release to levels similar to those seen with wild-type EVs apart from a higher level of release of IL-10. Purified pneumolysin induced release of only very low levels of cytokines in DCs, suggesting that the cytokine induction by EVs is mostly pneumolysin independent and therefore dependent upon other EV constituents such as membrane-bound lipoteichoic acids and lipoproteins (see Table S2 in the supplemental material), known TLR2 agonists (24).

Complement deposition has an essential role in the clearance of S. pneumoniae from the host, since it promotes phagocytosis by immune cells (25), and pneumococci are able to evade this mechanism through both the thick capsule and specific surface proteins (26). It is known that membrane vesicles can act as decoys for bacteriophages or antimicrobial peptides (4, 27) or can actively degrade complement proteins through proteases (28). Moreover, OMVs from Moraxella catarrhalis are known to possess the proteins UspA1/A2, which are able to bind C3 and protect Haemophilus influenzae from complement-mediated killing *in vitro* (29). Here we provide evidence that pneumococcal EVs can also bind key proteins of the complement system such as C3, leading to the assembly of the final product of the cascade, C5b-9, representing the plasma membrane-spanning MAC. Virtually all EVs gave positive staining results for C3b and C5b-9, suggesting that nucleophilic targets and the plasma membrane bilayer are freely accessible. Finally, our data suggest that EVs might be relevant *in vivo* by binding complement factors in serum, leading to reduced bacterial interaction with complement receptors on phagocytic cells and thereby impairing opsonophagocytic killing.

In conclusion, we show that pneumococci produce EVs that we suggest represent outward buddings from the bacterial plasma membrane, leading to vesicles with a cytoplasmic cargo enriched for the major cytotoxin pneumolysin. EVs can be internalized by epithelial cells and DCs and can evoke proinflammatory responses that are pneumolysin independent. Furthermore, EVs bind complement proteins and enhance the ability of pneumococci to evade complement-mediated opsonophagocytosis. We suggest that pneumococcal EVs could be a potential mechanism for the delivery of virulence proteins such as pneumolysin and other immunomodulatory components into host cells. During early stages of pneumococcal infection, vesicles released by bacteria could promote an inflammatory environment in the lower airways, possibly contributing to the symptoms of pneumococcal invasive disease. During sepsis, production of EVs can be a potent way for S. pneumoniae to avoid complement deposition and phagocytosis-mediated killing.

## MATERIALS AND METHODS

### Bacterial strains and growth conditions.

Five strains of S. pneumoniae were used: T4 (TIGR4 of serotype 4) (30) and its isogenic mutants lacking pneumolysin (T4*Δply*) (12), the autolysin LytA (T4*ΔlytA*) (11), the adhesin PspC (strain T4*ΔpspC*) (kindly provided by Peter Mellroth), and the capsule (strain T4R) (31). Bacteria were grown in C + Y medium (pH 7.9 to 8.0) at 37°C. Growth was measured by monitoring the optical density (OD) at 600 nm.

### Isolation and purification of extracellular vesicles (EVs).

Pneumococci were grown in C + Y medium until an OD_600_ of 0.9 was reached and were pelleted using centrifugation (17,000 × *g* for 30 min at 4°C). Culture supernatants were filtered (Sarstedt) (0.22-μm pore size) and centrifuged (120,000 × *g* for 2 h at 4°C) to cause sedimentation of the vesicles. Vesicles were washed and resuspended in phosphate-buffered saline (PBS). Crude vesicle preparations were further purified by density gradient centrifugation using OptiPrep density gradient medium (Sigma). Pelleted EVs were adjusted to 50% (wt/vol) OptiPrep in a total volume of 2 ml and overlaid with one fraction of 30% (wt/vol) OptiPrep (9 ml) followed by a fraction of 5% (wt/vol) OptiPrep (3 ml). Gradients were centrifuged at 155,000 × *g* for 3 h at 4°C, and the first 4 ml on top (containing the EVs) was collected. EVs were washed with PBS and stored at −80°C.

### Electron microscopy.

T4R was grown in C + Y medium until an OD_600_ of 0.4 was reached, harvested by centrifugation for 10 min at 4,000 × *g* and 4°C, and resuspended in PBS. Glow-discharged carbon-coated grids (Oxford Instruments, United Kingdom) were incubated for 1 min with a drop of bacterial solution or purified EVs and negatively stained with 2% uranyl actetate in water. Specimens were examined on a FEI CM120 microscope operated at 80 kV. Images were collected with a side-mounted MegaView III camera (Olympus Soft Imaging solutions).

### Atomic force microscopy.

Five microliters of EVs were placed onto freshly cleaved mica (Goodfellow Cambridge Ltd.), blot dried, and placed into a desiccator for a period of at least 2 h. Imaging was performed on a Nanoscope IIIa atomic force microscope (Digital Instruments) using tapping mode with standard silicon cantilevers oscillating at resonant frequency (270 to 305 kHz). Images were collected at a scan rate of 0.8 to 1.5 Hz, depending on sample number and the size of the scan. The final images were fitted in both axes and presented in a surface plot of the height mode.

### SDS-PAGE and Western blotting.

For detection of C3 binding to EVs, vesicles were incubated with 20% human serum (Sigma) for 2 h at 37°C. After washing, EVs were resuspended in PBS. The total amount of protein present in purified and serum-treated EVs was quantified using a Pierce bicinchoninic acid (BCA) protein assay kit (Life Technologies, Inc.). Equal amounts of total protein were resolved by SDS-PAGE using 4% to 12% bis-Tris gels (Life Technologies, Inc.) and stained with Coomassie blue. For immunoblotting, proteins on the gels were transferred to polyvinylidene difluoride (PVDF) membranes, blocked with 5% skim milk–PBS–0.1% Tween 20, and incubated with antibodies as indicated. For detection, the following antibodies were used: mouse monoclonal pneumolysin antibody (Abcam, Inc.; final dilution, 1:500); rabbit polyclonal antibodies against GAPDH (1:2,000), LytA (1:2,000) (11), RrgB (1:1,000), PsaA (1:25,000), and PhtD (1:25,000); and mouse polyclonal antibodies against PspC (1:1,000) and SrtA (1:500). C3 was detected with goat polyclonal anti-C3 (Calbiochem) (1:150). Anti-mouse IgG, anti-rabbit IgG, and anti-goat IgG conjugated to horseradish peroxidase (GE HealthCare) were used as secondary antibodies (1:10,000). Blots were developed with an Amersham ECL Plus Western blotting detection system (GE HealthCare). Pneumolysin in EVs was quantified with ImageJ in respect to purified pneumolysin.

### Tandem mass spectrometry.

EV preparations were lysed in a urea-containing buffer according to a standard operating procedure. The total protein concentration was measured using the Bradford protein assay with bovine serum albumin (BSA) as the standard. Aliquots corresponding to 15 μg of protein from each sample were used for digestion. Proteins in EV samples were reduced, alkylated, and subjected to in-solution digestion by trypsin according to a standard operating procedure. The samples were purified by Pierce C_18_ spin columns (Thermo Scientific), dried, and resolved in 0.1% formic acid. The resulting peptides were separated in the reverse phase on a C_18_ column and electrosprayed on line using a Q Exactive Plus mass spectrometer (Thermo Finnigan). Tandem mass spectrometry was performed applying higher-energy collisional dissociation (HCD).

Database searches were made using the Sequest algorithm embedded in Proteome discoverer 1.4 (Thermo Scientific) and a FASTA database, including proteins from TIGR4 downloaded from the UniProtKB database. The search criteria for protein identification were set to at least two matching peptides of 95% confidence level per protein. Only proteins with a Sequest score above 20 were considered for analysis, to avoid the possibility of false positives. The protein sequences were queried for the presence of transmembrane (TM) domains using SCAMPI2 (32) and TMHMM 2.0 (http://www.cbs.dtu.dk/services/TMHMM) and type I and type II signal peptidases using SignalP 4.1 (33) as well as LipoP 1.0 (34), and choline-binding/LPXTG cell wall-anchoring domains. Subcellular localization of the proteins was predicted as previously described (35).

For analysis of complement proteins, gel bands of EVs incubated with human serum were reduced, alkylated, and subjected to in-gel digestion by trypsin according to standard operating procedures. Samples were dried and resolved in 15 μl 0.1% formic acid, and peptides were separated in the reverse phase on a C_18_ column and electrosprayed on line using an LTQ-Orbitrap Velos Pro ETD mass spectrometer (Thermo Finnigan). Tandem mass spectrometry was performed applying collision-induced dissociation (CID). Database searches were performed using the Sequest algorithm embedded in Proteome discoverer 1.4 (Thermo Fisher Scientific) against a FASTA database containing the reference proteomes for Homo sapiens downloaded from the UniProtKB database.

### Measurement of cell toxicity of A549 lung epithelial cells.

A459 lung epithelial cells (13) were grown and maintained at 37°C in 5% CO_2_ with RPMI medium (Gibco) supplemented with 10% (vol/vol) fetal bovine serum (FBS) (HyClone). To assess EV-associated cytotoxic effects, 0.6 × 10^6^ A549 cells were seeded in 6-well plates and incubated overnight at 37°C. Cells were then washed with PBS and incubated for 24 h with medium containing EVs or purified pneumolysin at the indicated concentrations. Cells were labeled with eFluor 780 fixable viability dye (EBioscience) (1:50,000) for 30 min at 4°C in the dark in the presence or absence of NP-40 detergent (Sigma) (0.02%) as a positive control and fixed with 4% paraformaldehyde (PFA) for 30 min. Next, cells were gently scraped into PBS–1% FBS and analyzed in a Gallios flow cytometer (Beckman Coulter, Inc.).

### Immunofluorescence microscopy of A549 cells and dendritic cells (DCs).

A549 cells (6.25 × 10^4^) or DCs (7 × 10^4^) were seeded in 24-well plates with coverslips and incubated at 37°C overnight (A549 cells) or for 2 h (DCs). For DCs, poly-l-lysine-pretreated coverslips were used. Cells were washed with PBS and incubated for 6 h (A549 cells) or 2 h (DCs) with medium containing EVs at the indicated concentrations. After washing, the cells were fixed with 4% PFA for 30 min and permeabilized with 1% Triton X-100–PBS for 5 min (A549 cells) or 15 min (DCs). EVs were detected using mouse monoclonal anti-Ply (Abcam Inc.) (1:200) and Alexa Fluor 488-conjugated goat anti-mouse IgG antibody (Life Technologies, Inc.) (1:1,000). Actin cytoskeleton was stained with Alexa Fluor 594 phalloidin (Life Technologies, Inc., 1:40) for 1 h. Coverslips were mounted onto microscope slides with Vectashield (Vector Laboratories, Inc.). Images were acquired with a DeltaVision microscope equipped with a 100× objective. Quick-projection images of approximately 20 z-stacks were taken. Orthogonal views were used to quantify cells with intracellular vesicles.

### Isolation and differentiation of human monocyte-derived DCs.

DCs were isolated using RosetteSep human monocyte enrichment cocktail (StemCell Technologies) according to the manufacturer’s instructions. In brief, blood from buffy coats from healthy donors was incubated for 20 min with RosetteSep human monocyte enrichment cocktail (StemCell Technologies), layered on top of Ficoll-Paque Plus (GE HealthCare), and centrifuged at 1,200 × *g* for 20 min without acceleration or braking. The monocyte-containing layer was recovered, and cells were washed with PBS and passed through a 100-μm-pore-size cell strainer. Monocytes were then differentiated for 6 days in RPMI medium containing 10% FBS supplemented with 37.5 ng/ml of granulocyte-macrophage colony-stimulating factor (GM-CSF) (PeproTech) and 37.5 ng/ml of interleukin-4 (IL-4) (PeproTech), with changing of the medium after 4 days. For experiments, cells were resuspended in RPMI medium containing 10% FBS.

### Toxicity and apoptosis assay of DCs.

Cells (6 × 10^5^) were seeded in 96-well plates and incubated with RPMI medium containing 10% FBS and EVs at the indicated concentrations or with T4R at a multiplicity of infection (MOI) of 20, as a positive control, for 24 h. Gentamicin (Sigma) (100 μg/ml) was added after 1 h of incubation to stop bacterial growth. Before staining was performed, cells were washed with PBS and annexin V binding buffer (BD Pharmingen). Staining was performed with eFluor 780 fixable viability dye (EBioscience) (1:50,000) and fluorescein isothiocyanate (FITC) annexin V (BD Pharmingen) (1:20) for 30 min at 4°C, followed by washing with annexin buffer. Labeled cells were fixed in 4% PFA for 30 min, resuspended in PBS–1% FBS, and analyzed in a Gallios flow cytometer (Beckman Coulter, Inc.).

### Activation assay of DCs.

DCs (6 × 10^5^) were seeded in 96-well plates and incubated in RPMI medium containing 10% FBS with EVs at the indicated concentrations, or with 1 μg/ml lipopolysaccharide (LPS) (Sigma) as a positive control, for 24 h. Cells were stained with phycoerythrin (PE) mouse anti-human CD86 (BD Pharmingen) and with PE-Cy5 mouse anti-human HLA-DR (BD Pharmingen) for 20 min at 4°C, washed with PBS, and resuspended in PBS–1% FBS. Labeled cells were analyzed in a Gallios flow cytometer (Beckman Coulter, Inc.).

### Quantification of cytokines.

Cytokines (IL-6, IL-8, IL-10, IL-1β, IL-12p70, and TNF) were assessed in cell-free supernatants of 10^5^ DCs by enzyme-linked immunosorbent assay (ELISA), using commercially available BD OptEIA kits from BD Biosciences. Cells were incubated with EVs, with corresponding amounts of purified pneumolysin at the indicated concentrations, or with T4R (MOI of 20) or LPS (1 µg/ml) for 24 h.

### Complement binding using ELISA.

Plates with 96 wells were coated overnight at 4°C with 1 μg/ml of EVs in 0.1 M sodium-carbonate buffer (pH 9.5). Nonspecific binding was blocked with 1% BSA for 1 h, and plates were incubated with human serum at the indicated concentrations for 2 h. Complement binding was detected with goat polyclonal anti-C3 (Calbiochem) (1:16,000), mouse monoclonal anti-C5b-9 (Santa Cruz) (1:500), or goat polyclonal anti-factor H (Calbiochem) (1:16,000). Signal amplification was obtained with anti-mouse IgG (1:4,000) or anti-goat IgG (1:16,000) conjugated to horseradish peroxidase (GE HealthCare). Plates were then incubated with a tetramethylbenzidine (TMB) substrate reagent set (BD Biosciences) for 10 min, and the reaction was stopped with 1 M H_3_PO_4_.

### Immunofluorescence microscopy of EVs.

EV preparations were diluted in PBS, placed on a microscope slide, and dried. After washing, EVs were incubated with 20% human serum (Sigma) for 2 h. Slides were washed and incubated with Nile red (1:100) for 1 h. EVs and complement proteins were detected with mouse monoclonal anti-Ply (Abcam Inc.) (1:150), goat polyclonal anti-factor H (1:150) (Calbiochem), goat polyclonal anti-C3 (1:150) (Calbiochem), or mouse monoclonal anti-C5b-9 (1:150) (Santa Cruz). Addition of anti-Ply antibodies was followed by incubation with Alexa Fluor 350-conjugated donkey anti-mouse IgG antibody (Life Technologies, Inc.). For complement proteins, Alexa Fluor 488-conjugated goat anti-mouse IgG antibody (Life Technologies, Inc.) or Alexa Fluor 488-conjugated donkey anti-goat IgG antibody (Life Technologies, Inc.) (1:500) was used. Vectashield (Vector Laboratories, Inc.) and coverslips were then placed on top of the samples. Images were acquired with a DeltaVision microscope equipped with a 100× objective.

### Opsonophagocytosis assay using human THP-1 macrophages.

Human THP-1 cells were grown and maintained at 37°C in 5% CO_2_ and RPMI medium (Gibco) supplemented with 10% (vol/vol) FBS (HyClone). Cells (5 × 10^5^) were seeded in 24-well plates and differentiated with 20 ng/ml of phorbol 12-myristate 13-acetate (PMA; Sigma) for 48 h. Human serum (Sigma) was incubated with or without EVs (50 μg/ml) for 2 h at 37°C. S. pneumoniae was then incubated with 20% serum for 30 min at 37°C. THP-1 cells were washed to remove nonadherent cells, and S. pneumoniae was added for 1.5 h at 37°C at a MOI of 60. Cells were washed with PBS to remove unbound bacteria and lysed in 1% saponin for 15 min at 37°C. The lysates were serially diluted in PBS and plated on blood agar plates. To evaluate phagocytosis, 750 µg/ml of gentamicin was added to kill extracellular bacteria for 15 min at 37°C. Levels of adhered bacteria were calculated by subtracting the number of CFU present after gentamicin treatment from the total amount of cell-associated CFU. The adhesion ratio was calculated by dividing the total amount of cell-associated CFU by the amount of CFU present in the medium. The uptake ratio was calculated by dividing the number of bound bacteria by the total amount of cell-associated CFU.

### Opsonophagocytosis killing assay (OPKA).

Human HL-60 (ECACC 98070106) promyelocyte cells were grown at 37°C in 5% CO_2_ and RPMI medium (Gibco) supplemented with 10% (vol/vol) FBS (HyClone) and differentiated for 5 days with 1.5% dimethyl sulfoxide (DMSO) (Sigma). OPKAs were performed similarly to what was previously described (36). Briefly, human serum or heat-inactivated serum was added to 96-well plates. Bacteria were washed in opsonization buffer, i.e., Hanks’ balanced salt solution with Ca^2+^ and Mg^2+^ (Gibco) supplemented with 0.1% gelatin (Sigma) and 10% FBS (HyClone), and 1,200 CFU/well was incubated with serum at room temperature for 30 min. Differentiated HL-60 cells (4 × 10^5^ per well) were added, and plates were incubated for 1 h at 37°C in 5% CO_2_ with slow shaking. Bacterial survival was calculated by dividing the number of bacteria present at the end of the assay by the number of bacteria present before adding cells. Results were normalized to the number of bacteria present in wells without HL-60 cells.

### Hemolysis assays.

Purified EVs or recombinant pneumolysin was incubated in 96-well plates with blood from buffy coats (diluted 1:50 in PBS containing 1 mM dithiothreitol [DTT]) for 1 h at 37°C. After 50 min, 1% Triton X-100–PBS was added to the positive-control wells in order to lyse all erythrocytes. After 10 min, plates were spun at 400 × *g* for 15 min at 4°C, the supernatant was transferred to an optical plate, and the optical density was measured at 540 nm.

### Statistical analysis.

Data were statistically analyzed by one-way analysis of variance (ANOVA) test and Dunnett’s posttest. Statistically significant data were defined as follows: *, *P* ≤ 0.05; **, *P* ≤ 0.01; ***, *P* ≤ 0.001; ****, *P* ≤ 0.0001.
