# Supplementary figures and images for: Immunomodulatory Effects of Pneumococcal Extracellular Vesicles on Cellular and Humoral Host Defenses
Source: mBio. 2018 Apr 10;9(2):e00559-18. doi: 10.1128/mBio.00559-18 (PMC5893880; doi:10.1128/mBio.00559-18)

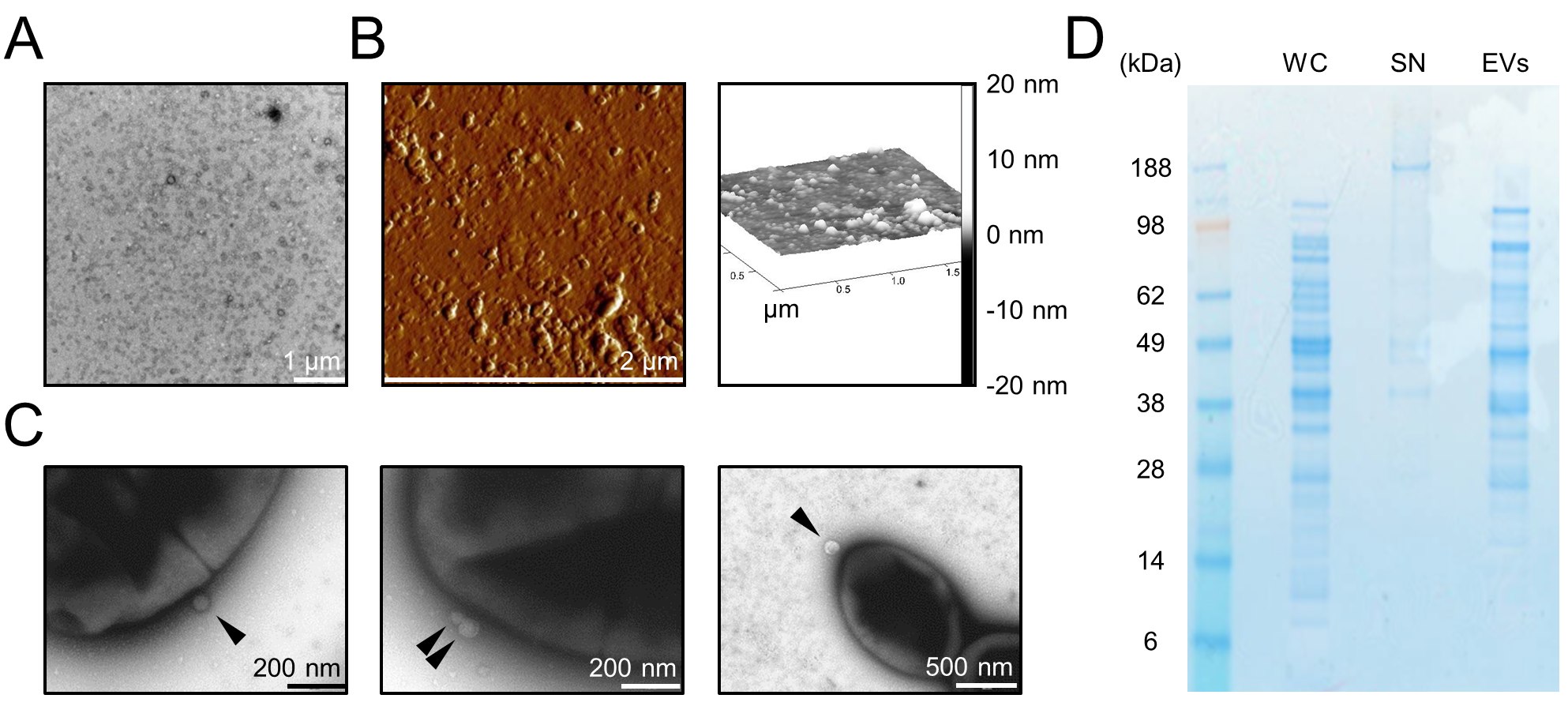

Supplement: FIG S1 [file mbo002183824sf1.tif]

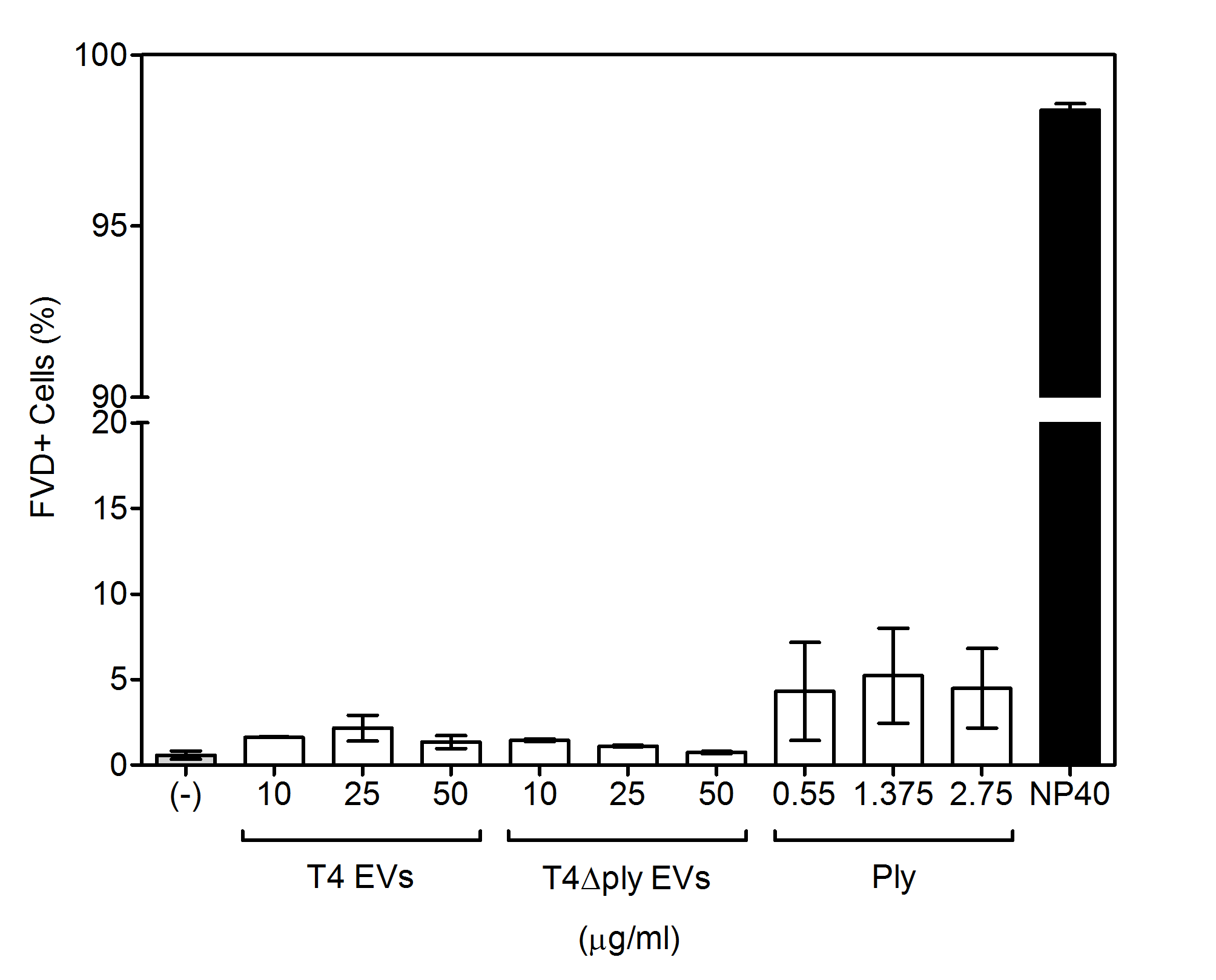

Supplement: FIG S2 [file mbo002183824sf2.tif]

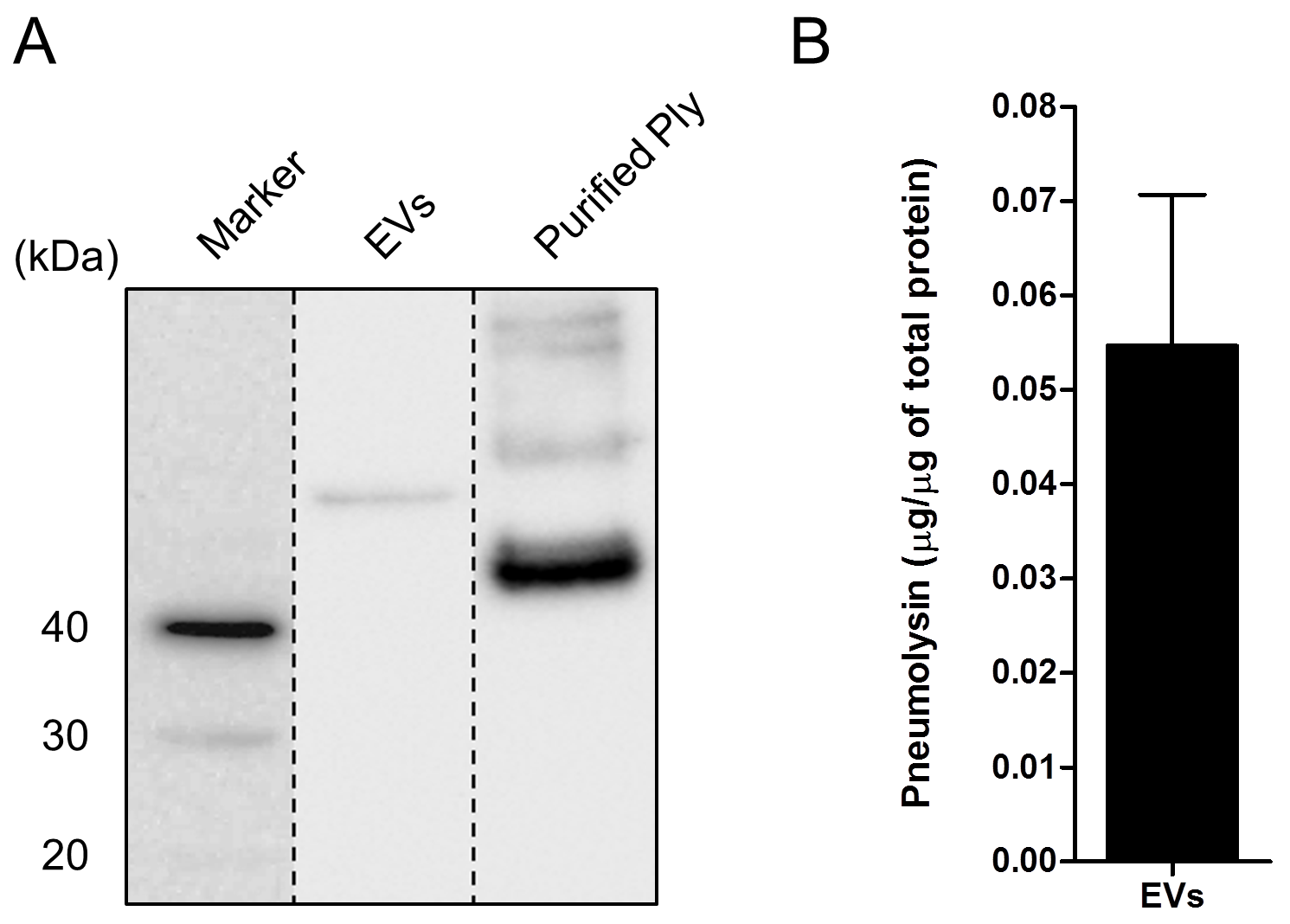

Supplement: FIG S3 [file mbo002183824sf3.tif]

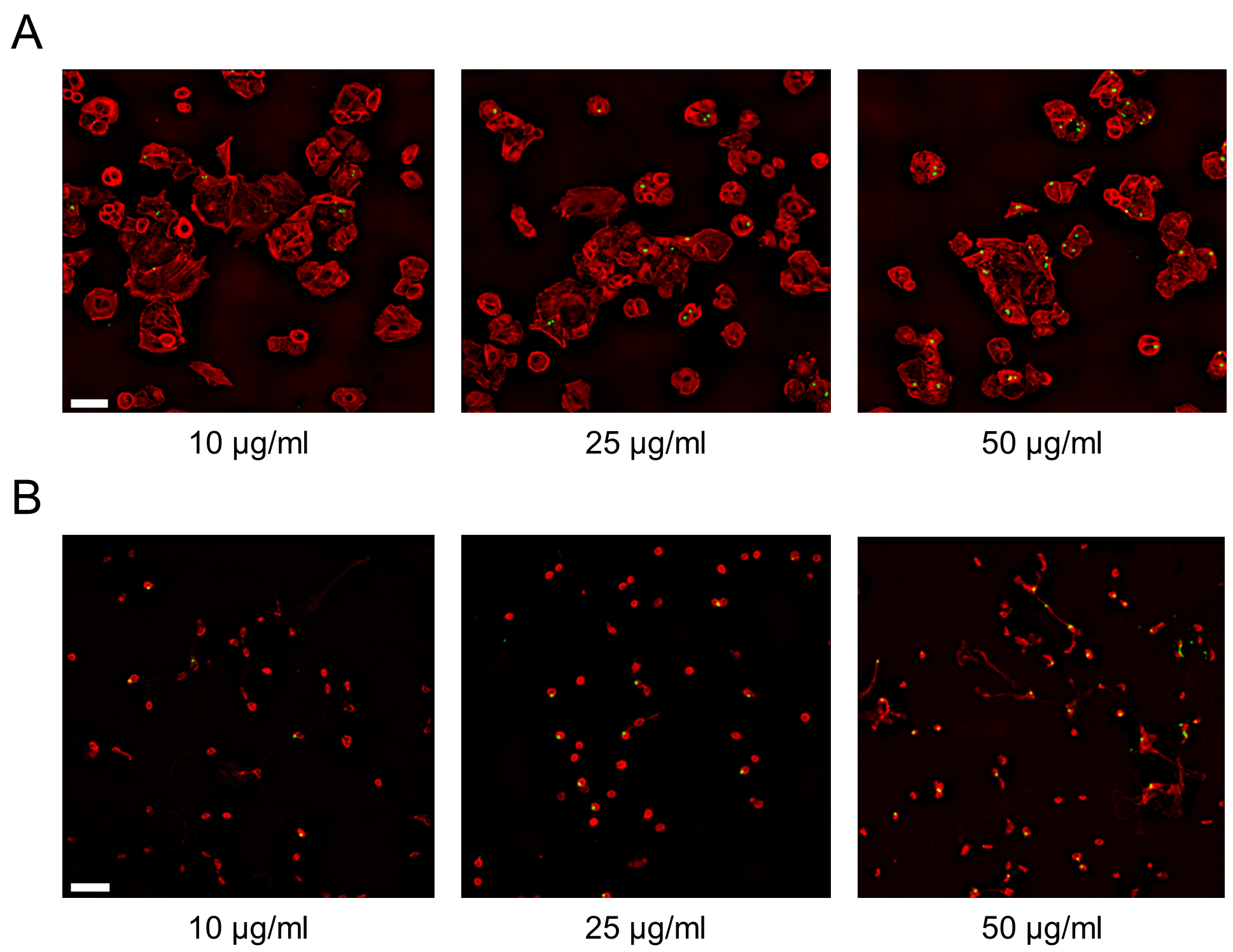

Supplement: FIG S4 [file mbo002183824sf4.tif]

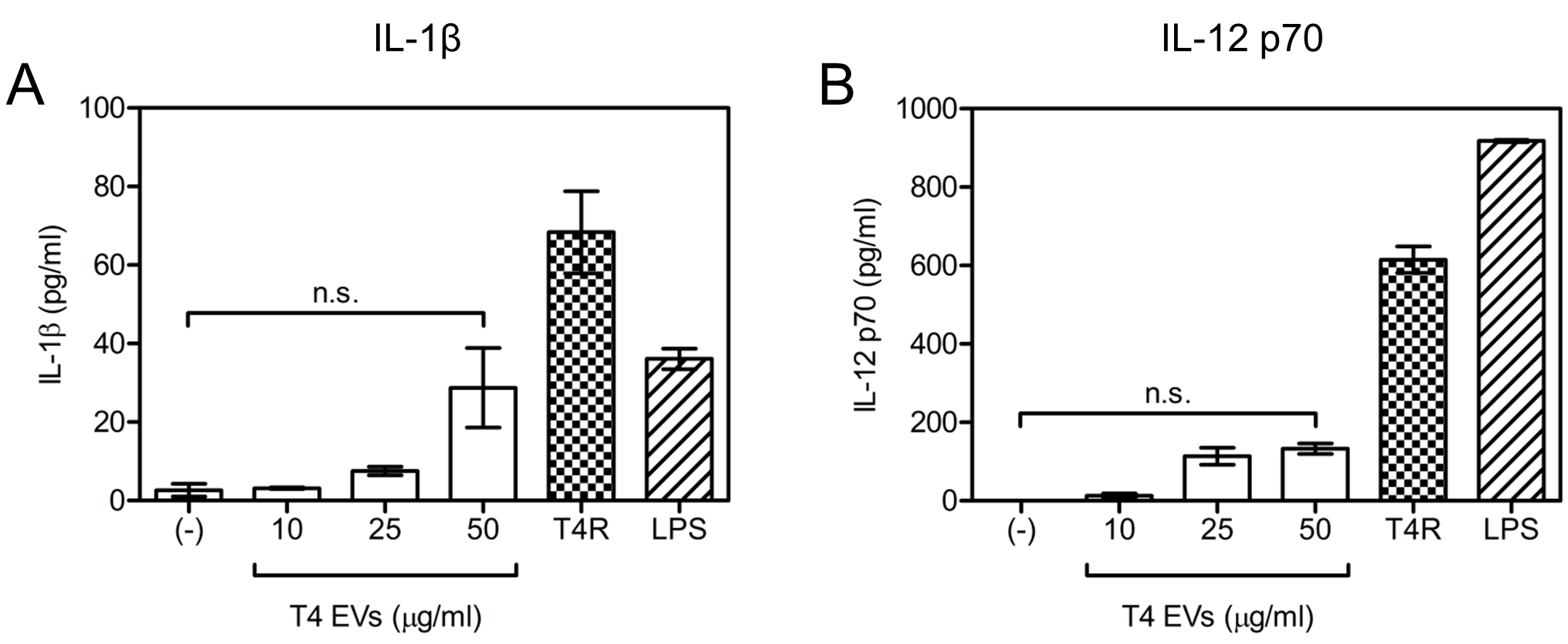

Supplement: FIG S5 [file mbo002183824sf5.tif]

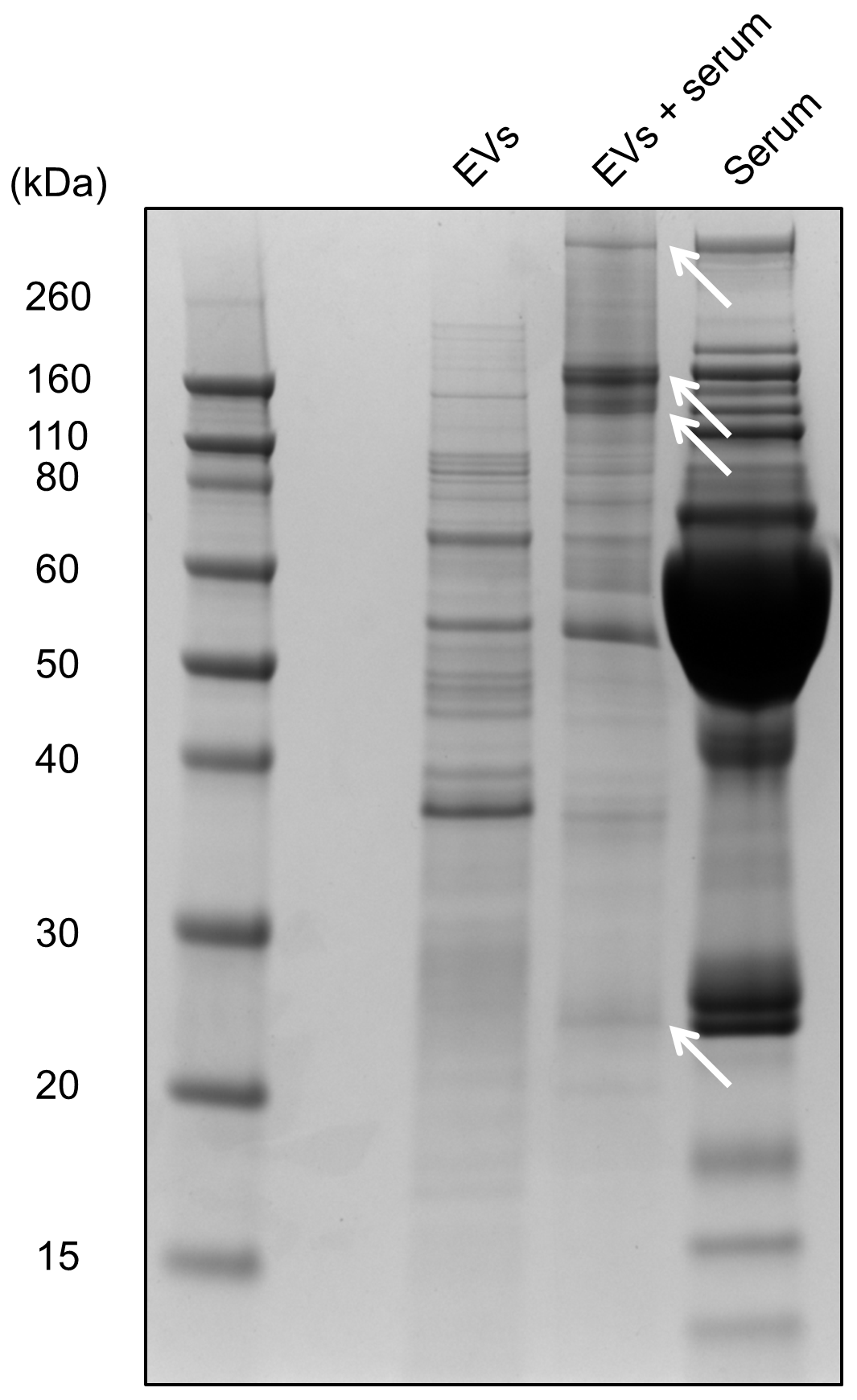

Supplement: FIG S6 [file mbo002183824sf6.tif]

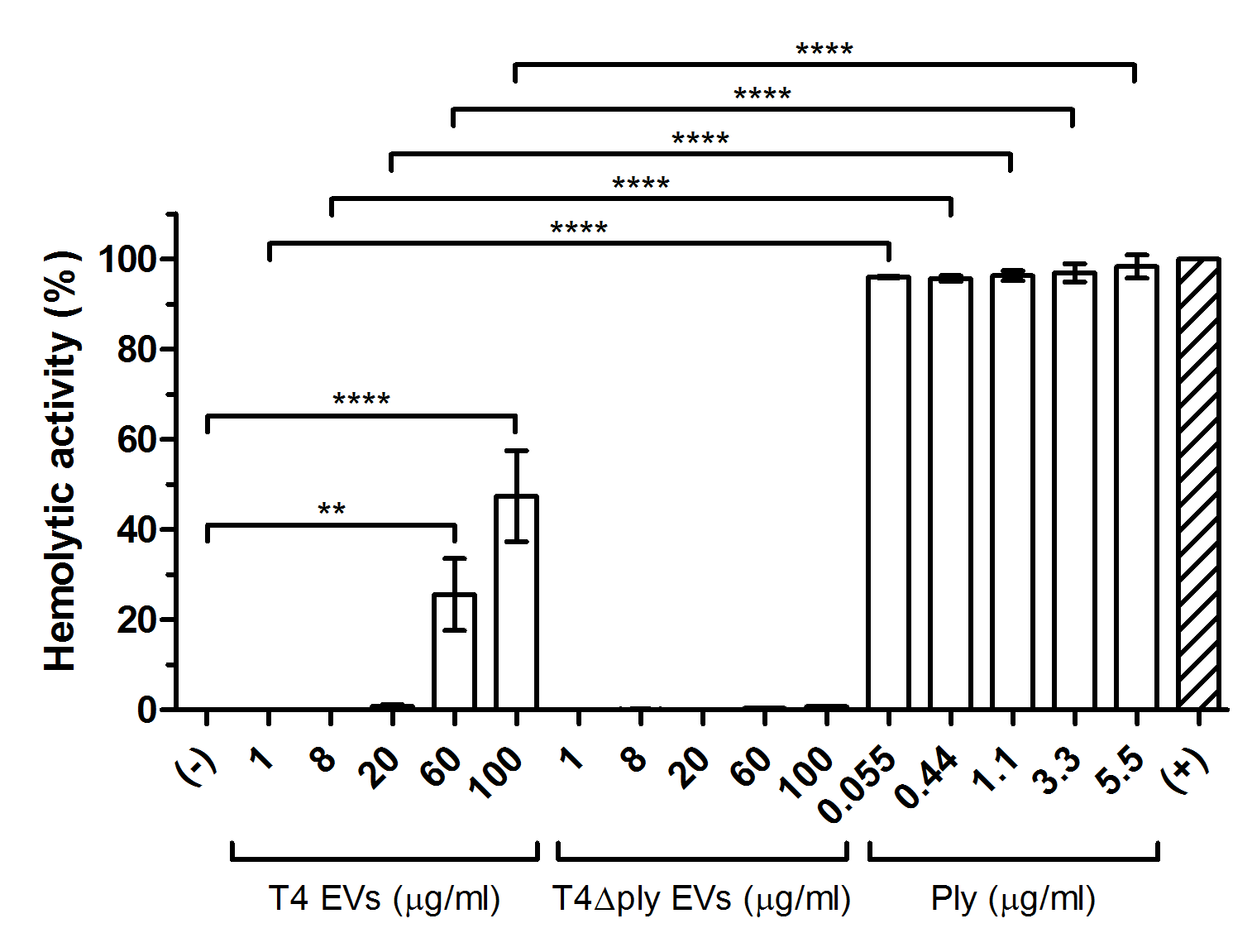

Supplement: FIG S7 [file mbo002183824sf7.tif]
