# Supplementary material for: Immunomodulatory Effects of Pneumococcal Extracellular Vesicles on Cellular and Humoral Host Defenses
Source: mBio. 2018 Apr 10;9(2):e00559-18. doi: 10.1128/mBio.00559-18 (PMC5893880; doi:10.1128/mBio.00559-18)
Supplement: TABLE S1 [file mbo002183824st1.docx]

**Table S1.** **Mass spectrometry identification of pneumococcal proteins present in EVs and their topology prediction**

| **Entry** | **Coverage** | **#Peptides** | **#AAs** | **Score** | **Gene name** | **Locus- tag** | **Choline-binding domain (CBD) /LPxTG proteins** | **Prediction of SPII (LipoP) and SPI (SignalP)** | **Prediction of TM domains (SCAMPI)** | **Prediction of TM domains (TMHMM)** | **Prediction of sub-cellular localization** |
| --- | --- | --- | --- | --- | --- | --- | --- | --- | --- | --- | --- |
| A0A0H2XFA2 | 82.45 | 59 | 490 | 1620.74 | lytC | SP_1573 | CBD | SPI | - | - | Cell wall |
| Q9L7Q2 | 55.82 | 91 | 1906 | 1166.89 | zmpB | SP_0664 | N-LPxTG | SPI | TM(1) | TM(2) | Cell wall |
| Q97T80 | 58.3 | 87 | 1856 | 1012.57 | zmpC | SP_0071 | N-LPxTG | SPI | TM(1) | TM(2) | Cell wall |
| A0A0H2UP55 | 42.1 | 73 | 2140 | 715.85 |  | SP_0641 | C-LPxTG | SPI | TM(1) | TM(1) | Cell wall |
| A0A0H2UMZ8 | 36.42 | 28 | 744 | 407.46 | pspA | SP_0117 | CBD | SPI | - | - | Cell wall |
| A0A0H2UPM5 | 41.79 | 28 | 627 | 381.01 | cbpE | SP_0930 | CBD | SPI | - | - | Cell wall |
| A0A0H2US50 | 47.47 | 31 | 693 | 320.12 | pspC | SP_2190 | CBD | SPI | - | - | Cell wall |
| P59205 | 43.77 | 20 | 658 | 217.64 | lytB | SP_0965 | CBD | SPI | - | - | Cell wall |
| A0A0H2USF9 | 42.19 | 22 | 621 | 215.44 | pcpA | SP_2136 | CBD | SPI | - | - | Cell wall |
| A0A0H2UP31 | 59.34 | 16 | 332 | 182.68 | cbpL | SP_0667 | CBD | SPI | - | - | Cell wall |
| A0A0H2UNN3 | 40.88 | 11 | 340 | 140.1 | cbpC | SP_0377 | CBD | SPI | - | - | Cell wall |
| A0A0H2UNH9 | 32.35 | 12 | 340 | 114.44 | cbpF | SP_0391 | CBD | SPI | - | - | Cell wall |
| Q2MGH6 | 15.73 | 18 | 1767 | 93.68 |  | SP_0368 | C-LPxTG | SPI | TM(1) | - | Cell wall |
| Q97SE4 | 32.3 | 12 | 514 | 70.62 | prfC | SP_0439 | C-LPxTG | - | - | - | Cell wall |
| A0A0H2UNH0 | 16.87 | 6 | 332 | 44.57 | cbpJ | SP_0378 | CBD | SPI | - | - | Cell wall |
| P06653 | 26.1 | 6 | 318 | 43.37 | lytA | SP_1937 | CBD | - | - | - | Cell wall |
| A0A0H2URT2 | 73.39 | 64 | 883 | 1364.43 |  | SP_2026 | - | - | - | - | Cytosolic |
| Q97NQ8 | 59.59 | 75 | 1225 | 936.07 | rpoC | SP_1960 | - | - | - | - | Cytosolic |
| P64022 | 75.61 | 46 | 693 | 803.77 | fusA | SP_0273 | - | - | - | - | Cytosolic |
| P0C2J9 | 92.99 | 46 | 471 | 753.38 | ply | SP_1923 | - | - | - | - | Cytosolic |
| P64030 | 68.84 | 32 | 398 | 684.61 | tuf | SP_1489 | - | - | - | - | Cytosolic |
| Q97NQ7 | 66.42 | 61 | 1203 | 683.01 | rpoB | SP_1961 | - | - | - | - | Cytosolic |
| Q97SV2 | 62.45 | 19 | 277 | 575.15 | rplB | SP_0212 | - | - | - | - | Cytosolic |
| Q97SV5 | 59.62 | 17 | 208 | 571.92 | rplC | SP_0209 | - | - | - | - | Cytosolic |
| A0A0H2US80 | 88.36 | 26 | 335 | 546.25 | gap | SP_2012 | - | - | - | - | Cytosolic |
| Q97PD6 | 64.87 | 46 | 837 | 501.57 | secA1 | SP_1702 | - | - | - | - | Cytosolic |
| Q54970 | 61.59 | 38 | 591 | 482.11 | spxB | SP_0730 | - | - | - | - | Cytosolic |
| P0A4C3 | 72.35 | 20 | 217 | 427.97 | rpsC | SP_0215 | - | - | - | - | Cytosolic |
| P0A475 | 54.74 | 11 | 137 | 354.71 | rplP | SP_0216 | - | - | - | - | Cytosolic |
| P66112 | 51.26 | 9 | 119 | 346.49 | rplT | SP_0961 | - | - | - | - | Cytosolic |
| Q97SV4 | 56.04 | 14 | 207 | 345.01 | rplD | SP_0210 | - | - | - | - | Cytosolic |
| Q97QS2 | 58.06 | 22 | 434 | 315.73 | eno | SP_1128 | - | - | - | - | Cytosolic |
| Q97S57 | 49.58 | 34 | 958 | 315.37 | infB | SP_0556 | - | - | - | - | Cytosolic |
| P66359 | 55.12 | 10 | 127 | 299.97 | rpsK | SP_0235 | - | - | - | - | Cytosolic |
| Q97PT6 | 68.8 | 24 | 468 | 285.18 | atpD | SP_1508 | - | - | - | - | Cytosolic |
| A0A0H2UN99 | 60.24 | 18 | 332 | 279.63 | manL | SP_0284 | - | - | - | - | Cytosolic |
| P65887 | 62.62 | 25 | 428 | 243.93 | purA | SP_0019 | - | - | - | - | Cytosolic |
| Q97NQ0 | 68.79 | 19 | 330 | 230.07 | asnA | SP_1970 | - | - | - | - | Cytosolic |
| Q97SV1 | 86.67 | 12 | 180 | 229.35 | rplE | SP_0221 | - | - | - | - | Cytosolic |
| P66907 | 42.63 | 9 | 380 | 223.83 | tgt | SP_2058 | - | - | - | - | Cytosolic |
| A0A0H2UNL5 | 49.35 | 27 | 774 | 216.35 | pfl | SP_0459 | - | - | - | - | Cytosolic |
| P0A2U8 | 49.58 | 19 | 355 | 215.01 | amiE | SP_1888 | - | - | - | - | Cytosolic |
| A0A0H2UPG5 | 56.25 | 22 | 400 | 204.01 | rpsA | SP_0862 | - | - | - | - | Cytosolic |
| P0A4A7 | 58.39 | 11 | 137 | 202.86 | rpsL | SP_0271 | - | - | - | - | Cytosolic |
| Q97N56 | 69.11 | 20 | 259 | 201.44 | rpsB | SP_2215 | - | - | - | - | Cytosolic |
| Q97RC6 | 48.66 | 14 | 335 | 201.21 | pfkA | SP_0896 | - | - | - | - | Cytosolic |
| A0A0H2URS4 | 60.64 | 17 | 404 | 197.5 |  | SP_1994 | - | - | - | - | Cytosolic |
| A0A0H2UQC2 | 56.41 | 20 | 523 | 188.15 | ffh | SP_1287 | - | - | - | - | Cytosolic |
| P0A3M9 | 47.26 | 14 | 328 | 180.34 | ldh | SP_1220 | - | - | - | - | Cytosolic |
| Q97PT4 | 39.72 | 17 | 501 | 180.17 | atpA | SP_1510 | - | - | - | - | Cytosolic |
| A0A0H2UQ97 | 67.48 | 9 | 246 | 175.25 |  | SP_1242 | - | - | - | - | Cytosolic |
| Q97QC6 | 66.09 | 14 | 115 | 173.94 | rplS | SP_1293 | - | - | - | - | Cytosolic |
| A0A0H2US83 | 63.14 | 18 | 274 | 172.57 |  | SP_2156 | - | - | - | - | Cytosolic |
| A0A0H2UNS2 | 36.4 | 14 | 555 | 169.45 |  | SP_0443 | - | - | - | - | Cytosolic |
| A0A0H2UQW6 | 59.04 | 16 | 376 | 168.92 | msmK | SP_1580 | - | - | - | - | Cytosolic |
| A0A0H2UNF0 | 85.81 | 15 | 148 | 165.86 | rplM | SP_0294 | - | - | - | - | Cytosolic |
| P18766 | 50 | 13 | 308 | 160.11 | amiF | SP_1887 | - | - | - | - | Cytosolic |
| A0A0H2UNJ4 | 42.39 | 16 | 394 | 155.34 | cps4L | SP_0360 | - | - | - | - | Cytosolic |
| P0CC08 | 55.21 | 15 | 288 | 154.74 | accD | SP_0426 | - | - | - | - | Cytosolic |
| A0A0H2UPF8 | 51.27 | 21 | 511 | 154.04 |  | SP_0846 | - | - | - | - | Cytosolic |
| A0A0H2UNJ0 | 50.28 | 17 | 360 | 153.5 |  | SP_0355 | - | - | - | - | Cytosolic |
| P72524 | 33.82 | 22 | 822 | 148.92 | gyrA | SP_1219 | - | - | - | - | Cytosolic |
| P66565 | 56.16 | 17 | 203 | 148.91 | rpsD | SP_0085 | - | - | - | - | Cytosolic |
| Q97SG0 | 57.78 | 18 | 424 | 144.79 | serS | SP_0411 | - | - | - | - | Cytosolic |
| A0A0H2UR32 | 51.79 | 20 | 419 | 144.46 | ftsZ | SP_1666 | - | - | - | - | Cytosolic |
| Q97Q34 | 64.04 | 17 | 267 | 144.16 | pstB2 | SP_1397 | - | - | - | - | Cytosolic |
| A0A0H2UNK1 | 47.25 | 14 | 455 | 141.81 | accC | SP_0425 | - | - | - | - | Cytosolic |
| Q97SR1 | 45.54 | 19 | 617 | 140.84 | proS | SP_0264 | - | - | - | - | Cytosolic |
| P0A4D7 | 40.84 | 17 | 524 | 138.63 | cshA | SP_1586 | - | - | - | - | Cytosolic |
| A0A0H2UPF2 | 44.44 | 17 | 513 | 136.66 |  | SP_0770 | - | - | - | - | Cytosolic |
| Q97PR0 | 43.62 | 17 | 447 | 136.28 | asnS | SP_1542 | - | - | - | - | Cytosolic |
| P22976 | 40.73 | 18 | 658 | 132.04 | tkt | SP_2030 | - | - | - | - | Cytosolic |
| A0A0H2UQZ4 | 34.2 | 16 | 459 | 132 | nox | SP_1469 | - | - | - | - | Cytosolic |
| P66095 | 52.4 | 12 | 229 | 128.44 | rplA | SP_0631 | - | - | - | - | Cytosolic |
| A0A0H2UPG6 | 55.65 | 12 | 230 | 127.75 | ftsE | SP_0756 | - | - | - | - | Cytosolic |
| A0A0H2USJ7 | 38.4 | 23 | 810 | 122.4 |  | SP_2194 | - | - | - | - | Cytosolic |
| P95830 | 50.79 | 15 | 378 | 120.38 | dnaJ | SP_0519 | - | - | - | - | Cytosolic |
| P63373 | 71.03 | 13 | 252 | 116.79 | pstB1 | SP_1396 | - | - | - | - | Cytosolic |
| A0A0H2UP66 | 62.96 | 16 | 378 | 110.09 | lctO-2 | SP_0715 | - | - | - | - | Cytosolic |
| P0A4S1 | 49.15 | 9 | 293 | 109 | fba | SP_0605 | - | - | - | - | Cytosolic |
| Q97SI9 | 60.55 | 7 | 109 | 107.21 | gpsB | SP_0372 | - | - | - | - | Cytosolic |
| P63413 | 36.36 | 12 | 396 | 106.75 | ackA | SP_2044 | - | - | - | - | Cytosolic |
| Q97SQ4 | 39.74 | 8 | 156 | 103.23 | rpsG | SP_0272 | - | - | - | - | Cytosolic |
| Q97QK5 | 48.6 | 19 | 607 | 101.3 | lepA | SP_1200 | - | - | - | - | Cytosolic |
| P65239 | 39.75 | 11 | 322 | 100.54 | prs1 | SP_0027 | - | - | - | - | Cytosolic |
| P95829 | 36.41 | 14 | 607 | 100.51 | dnaK | SP_0517 | - | - | - | - | Cytosolic |
| A0A0H2UNK4 | 55.47 | 11 | 411 | 100.01 | fabF | SP_0422 | - | - | - | - | Cytosolic |
| Q97SQ9 | 36.05 | 13 | 602 | 92.56 | glmS | SP_0266 | - | - | - | - | Cytosolic |
| A0A0H2URD5 | 53.96 | 9 | 202 | 92.31 |  | SP_1804 | - | - | - | - | Cytosolic |
| Q97QE4 | 25.33 | 18 | 1058 | 90.95 | carB | SP_1275 | - | - | - | - | Cytosolic |
| A0A0H2UR24 | 40.04 | 11 | 457 | 90.76 | ftsA | SP_1667 | - | - | - | - | Cytosolic |
| Q97Q48 | 27.52 | 18 | 872 | 89.81 | alaS | SP_1383 | - | - | - | - | Cytosolic |
| A0A0H2UNF3 | 43.01 | 14 | 365 | 88.6 | cps4I | SP_0357 | - | - | - | - | Cytosolic |
| A0A0H2UPX2 | 36.47 | 13 | 425 | 87.93 |  | SP_1083 | - | - | - | - | Cytosolic |
| Q9FBB7 | 58.43 | 12 | 255 | 86.89 | accA | SP_0427 | - | - | - | - | Cytosolic |
| P67266 | 42.42 | 4 | 99 | 86.55 |  | SP_1102 | - | - | - | - | Cytosolic |
| A0A0H2UQ44 | 24.06 | 13 | 719 | 86.48 | nrdE | SP_1179 | - | - | - | - | Cytosolic |
| A0A0H2UR40 | 44.17 | 8 | 283 | 85.34 |  | SP_1674 | - | - | - | - | Cytosolic |
| P0A3Y3 | 50.87 | 10 | 230 | 84.95 | gpmA | SP_1655 | - | - | - | - | Cytosolic |
| A0A0H2US49 | 40.78 | 4 | 103 | 84.35 |  | SP_2102 | - | - | - | - | Cytosolic |
| Q97QX6 | 22.61 | 14 | 898 | 82.38 | ppc | SP_1068 | - | - | - | - | Cytosolic |
| P66419 | 50.56 | 8 | 89 | 78.12 | rpsN | SP_0222 | - | - | - | - | Cytosolic |
| P61182 | 61.4 | 8 | 114 | 77.58 | rplV | SP_0214 | - | - | - | - | Cytosolic |
| P66278 | 33.33 | 4 | 66 | 77.34 | rpmI | SP_0960 | - | - | - | - | Cytosolic |
| A0A0H2UN67 | 26.53 | 13 | 735 | 70.68 | nrdD | SP_0202 | - | - | - | - | Cytosolic |
| A0A0H2UNR2 | 47.33 | 7 | 243 | 68.48 | fabG | SP_0421 | - | - | - | - | Cytosolic |
| Q97S28 | 21.17 | 11 | 737 | 68.26 | pnp | SP_0588 | - | - | - | - | Cytosolic |
| A0A0H2UNQ5 | 21.65 | 7 | 448 | 65.38 | glnA | SP_0502 | - | - | - | - | Cytosolic |
| A0A0H2URX2 | 41.48 | 9 | 352 | 65.13 |  | SP_2055 | - | - | - | - | Cytosolic |
| P0A495 | 65.79 | 3 | 38 | 64.65 | rpmJ | SP_0233 | - | - | - | - | Cytosolic |
| Q97ND6 | 25.72 | 13 | 587 | 64.48 | aspS | SP_2114 | - | - | - | - | Cytosolic |
| P72525 | 18.1 | 10 | 823 | 63.42 | parC | SP_0855 | - | - | - | - | Cytosolic |
| A0A0H2UR16 | 23.11 | 12 | 740 | 63.08 | relA | SP_1645 | - | - | - | - | Cytosolic |
| A0A0H2UNF8 | 42.17 | 11 | 351 | 60.21 | cap4J | SP_0358 | - | - | - | - | Cytosolic |
| Q97RQ6 | 23.84 | 6 | 281 | 60.06 |  | SP_0742 | - | - | - | - | Cytosolic |
| A0A0H2UQB3 | 41.47 | 9 | 340 | 58.95 | tarJ | SP_1270 | - | - | - | - | Cytosolic |
| A0A0H2URM1 | 34.56 | 10 | 408 | 58.93 |  | SP_1837 | - | - | - | - | Cytosolic |
| Q97T09 | 40.23 | 9 | 353 | 57.99 | metN | SP_0151 | - | - | - | - | Cytosolic |
| P0A451 | 41.49 | 12 | 388 | 57.14 | recA | SP_1940 | - | - | - | - | Cytosolic |
| Q97SU7 | 51.12 | 5 | 178 | 56.93 | rplF | SP_0225 | - | - | - | - | Cytosolic |
| A0A0H2US09 | 18.49 | 9 | 687 | 56.54 |  | SP_2101 | - | - | - | - | Cytosolic |
| Q97SN4 | 32.31 | 6 | 130 | 56.22 | rpsI | SP_0295 | - | - | - | - | Cytosolic |
| P66392 | 51.24 | 7 | 121 | 56.15 | rpsM | SP_0234 | - | - | - | - | Cytosolic |
| A0A0H2UNZ5 | 23.51 | 11 | 553 | 55.98 | rnj | SP_0613 | - | - | - | - | Cytosolic |
| A0A0H2UR19 | 20.99 | 5 | 262 | 55.94 | divIVA | SP_1661 | - | - | - | - | Cytosolic |
| A0A0H2UNG2 | 43.52 | 12 | 409 | 55.75 | cps4K | SP_0359 | - | - | - | - | Cytosolic |
| Q97PI4 | 29.98 | 12 | 647 | 55.52 | thrS | SP_1631 | - | - | - | - | Cytosolic |
| Q97QW8 | 25.81 | 8 | 434 | 55.41 | obg | SP_1079 | - | - | - | - | Cytosolic |
| P0A4L9 | 20.52 | 10 | 648 | 54.97 | gyrB | SP_0806 | - | - | - | - | Cytosolic |
| Q97SJ8 | 33.2 | 11 | 494 | 54.78 |  | SP_0341 | - | - | - | - | Cytosolic |
| Q54869 | 24.16 | 10 | 563 | 54.43 | argS | SP_2078 | - | - | - | - | Cytosolic |
| P0A4B5 | 55.91 | 7 | 93 | 52.72 | rpsS | SP_0213 | - | - | - | - | Cytosolic |
| Q97SU3 | 23.29 | 4 | 146 | 52.09 | rplO | SP_0229 | - | - | - | - | Cytosolic |
| A0A0H2UP44 | 27.57 | 11 | 613 | 51.74 |  | SP_0681 | - | - | - | - | Cytosolic |
| A0A0H2UQJ0 | 41.6 | 9 | 262 | 49.97 | pck | SP_1269 | - | - | - | - | Cytosolic |
| P67595 | 31.96 | 8 | 341 | 48.09 | trpS | SP_2229 | - | - | - | - | Cytosolic |
| P66581 | 55.49 | 6 | 164 | 47.77 | rpsE | SP_0227 | - | - | - | - | Cytosolic |
| A0A0H2UPW1 | 16.33 | 10 | 784 | 47.19 | rnr | SP_0975 | - | - | - | - | Cytosolic |
| A0A0H2UPE7 | 21.03 | 7 | 447 | 46.86 | cshB | SP_0761 | - | - | - | - | Cytosolic |
| P0A3R1 | 22.65 | 9 | 649 | 46.48 | hexB | SP_0173 | - | - | - | - | Cytosolic |
| P0A3R3 | 18.48 | 11 | 844 | 45.87 | hexA | SP_2076 | - | - | - | - | Cytosolic |
| A0A0H2UNJ9 | 27.97 | 7 | 261 | 45.73 |  | SP_0415 | - | - | - | - | Cytosolic |
| P66708 | 45.98 | 9 | 311 | 45.6 | rpoA | SP_0236 | - | - | - | - | Cytosolic |
| A0A0H2USM1 | 19.51 | 7 | 492 | 45.08 | guaB | SP_2228 | - | - | - | - | Cytosolic |
| A0A0H2UNE6 | 42.22 | 7 | 270 | 44.36 |  | SP_0286 | - | - | - | - | Cytosolic |
| A0A0H2UR84 | 38.75 | 7 | 240 | 44.16 | psaB | SP_1648 | - | - | - | - | Cytosolic |
| A0A0H2UMX0 | 31.67 | 6 | 221 | 44.14 |  | SP_0079 | - | - | - | - | Cytosolic |
| Q97RS9 | 21.98 | 7 | 496 | 44.07 | lysS | SP_0713 | - | - | - | - | Cytosolic |
| P63791 | 27.8 | 8 | 410 | 42.54 | clpX | SP_1569 | - | - | - | - | Cytosolic |
| A0A0H2UQU1 | 37.33 | 7 | 217 | 42.45 |  | SP_1395 | - | - | - | - | Cytosolic |
| Q97R16 | 32.06 | 10 | 418 | 42.38 | glyA | SP_1024 | - | - | - | - | Cytosolic |
| A0A0H2URS9 | 38.99 | 9 | 336 | 42.33 | ccpA | SP_1999 | - | - | - | - | Cytosolic |
| A0A0H2UQU5 | 42.66 | 6 | 143 | 40.36 |  | SP_1545 | - | - | - | - | Cytosolic |
| Q97SU6 | 59.32 | 7 | 118 | 40.13 | rplR | SP_0226 | - | - | - | - | Cytosolic |
| A0A0H2UPR2 | 30.77 | 3 | 156 | 40.01 | argR | SP_0893 | - | - | - | - | Cytosolic |
| A0A0H2UPJ3 | 25.15 | 8 | 501 | 39.74 | pyk | SP_0897 | - | - | - | - | Cytosolic |
| Q97PM1 | 45.04 | 7 | 262 | 38.81 | codY | SP_1584 | - | - | - | - | Cytosolic |
| Q97S93 | 17.76 | 7 | 535 | 37.74 | pyrG | SP_0494 | - | - | - | - | Cytosolic |
| A0A0H2UPX1 | 20.71 | 9 | 763 | 37.7 | pcrA | SP_1087 | - | - | - | - | Cytosolic |
| P63544 | 51.76 | 6 | 170 | 37.59 | apt | SP_1577 | - | - | - | - | Cytosolic |
| P65241 | 18.18 | 5 | 319 | 37.57 | prs2 | SP_1095 | - | - | - | - | Cytosolic |
| Q97R12 | 19.52 | 7 | 543 | 37.11 |  | SP_1029 | - | - | - | - | Cytosolic |
| P35594 | 17.15 | 10 | 752 | 36.7 | clpE | SP_0820 | - | - | - | - | Cytosolic |
| P64072 | 53.33 | 8 | 195 | 36.63 | engB | SP_1568 | - | - | - | - | Cytosolic |
| Q97TC4 | 31.11 | 5 | 180 | 36.56 | hpt | SP_0012 | - | - | - | - | Cytosolic |
| A0A0H2UR17 | 37.8 | 4 | 209 | 36.45 |  | SP_1501 | - | - | - | - | Cytosolic |
| A0A0H2USA3 | 28.66 | 6 | 328 | 36.13 |  | SP_2040 | - | - | - | - | Cytosolic |
| P63742 | 19.32 | 7 | 502 | 36.12 | glpK | SP_2186 | - | - | - | - | Cytosolic |
| P64297 | 23.85 | 7 | 520 | 35.92 | guaA | SP_1445 | - | - | - | - | Cytosolic |
| Q97R84 | 28.38 | 7 | 444 | 35.81 | trmFO | SP_0943 | - | - | - | - | Cytosolic |
| A0A0H2UQY4 | 19.17 | 5 | 339 | 35.39 | galE-1 | SP_1607 | - | - | - | - | Cytosolic |
| A0A0H2UP95 | 41.53 | 6 | 236 | 35.16 | livF | SP_0753 | - | - | - | - | Cytosolic |
| A0A0H2URU0 | 30.74 | 10 | 462 | 35.12 |  | SP_1997 | - | - | - | - | Cytosolic |
| Q97S34 | 10.86 | 7 | 801 | 34.77 | pheT | SP_0581 | - | - | - | - | Cytosolic |
| Q97NQ4 | 31.38 | 9 | 427 | 34.05 | murA1 | SP_1966 | - | - | - | - | Cytosolic |
| A0A0H2UQM8 | 33.2 | 6 | 247 | 34.04 |  | SP_1460 | - | - | - | - | Cytosolic |
| Q97RQ3 | 44.5 | 6 | 209 | 33.91 | upp | SP_0745 | - | - | - | - | Cytosolic |
| A0A0H2UQ86 | 15.87 | 7 | 567 | 33.86 |  | SP_1161 | - | - | - | - | Cytosolic |
| P64062 | 24.77 | 8 | 436 | 33.49 | der | SP_1709 | - | - | - | - | Cytosolic |
| P65832 | 36.73 | 6 | 275 | 32.69 | purR | SP_1979 | - | - | - | - | Cytosolic |
| P63384 | 12.09 | 9 | 943 | 31.63 | uvrA | SP_0186 | - | - | - | - | Cytosolic |
| A0A0H2URQ9 | 33.88 | 8 | 242 | 31.53 | proV | SP_1861 | - | - | - | - | Cytosolic |
| Q9AHD2 | 40.97 | 8 | 227 | 31.44 | cpsD | SP_0349 | - | - | - | - | Cytosolic |
| O08397 | 23.84 | 8 | 453 | 31.17 | dnaA | SP_0001 | - | - | - | - | Cytosolic |
| Q97PT5 | 23.63 | 7 | 292 | 30.59 | atpG | SP_1509 | - | - | - | - | Cytosolic |
| A0A0H2UQN8 | 18.36 | 6 | 414 | 30.06 |  | SP_1472 | - | - | - | - | Cytosolic |
| A0A0H2UQ00 | 15.61 | 5 | 474 | 29.86 | gapN | SP_1119 | - | - | - | - | Cytosolic |
| Q97PW6 | 12.24 | 6 | 678 | 29.5 | glyS | SP_1474 | - | - | - | - | Cytosolic |
| A0A0H2UMU6 | 25.93 | 8 | 432 | 29.32 | purB | SP_0056 | - | - | - | - | Cytosolic |
| Q97TA6 | 12.42 | 3 | 330 | 28.21 | plsX | SP_0037 | - | - | - | - | Cytosolic |
| A0A0H2UN02 | 19.02 | 6 | 389 | 27.91 | araT | SP_0035 | - | - | - | - | Cytosolic |
| P0A3B7 | 23.99 | 5 | 346 | 27.48 | tsf | SP_2214 | - | - | - | - | Cytosolic |
| A0A0H2UNJ5 | 20.99 | 4 | 324 | 27.27 | fabK | SP_0419 | - | - | - | - | Cytosolic |
| Q97SG5 | 9.38 | 5 | 778 | 26.77 | mutS2 | SP_0406 | - | - | - | - | Cytosolic |
| A0A0H2UNU2 | 15.4 | 5 | 487 | 26.69 | hsdM | SP_0509 | - | - | - | - | Cytosolic |
| A0A0H2UNM5 | 12.78 | 4 | 454 | 26.54 |  | SP_0413 | - | - | - | - | Cytosolic |
| Q97S89 | 19.1 | 5 | 398 | 26.46 | pgk | SP_0499 | - | - | - | - | Cytosolic |
| A0A0H2UQF6 | 22.14 | 6 | 429 | 26.44 | ftsY | SP_1244 | - | - | - | - | Cytosolic |
| A0A0H2UR97 | 23.64 | 5 | 368 | 26.11 |  | SP_1749 | - | - | - | - | Cytosolic |
| P63733 | 20.06 | 6 | 359 | 25.98 | carA | SP_1276 | - | - | - | - | Cytosolic |
| A0A0H2UNB1 | 28.61 | 6 | 339 | 25.89 |  | SP_0285 | - | - | - | - | Cytosolic |
| P66339 | 48.04 | 4 | 102 | 25.74 | rpsJ | SP_0208 | - | - | - | - | Cytosolic |
| P0A335 | 13.52 | 6 | 540 | 25.62 | groL | SP_1906 | - | - | - | - | Cytosolic |
| A0A0H2UQV3 | 41.41 | 3 | 128 | 25.55 |  | SP_1558 | - | - | - | - | Cytosolic |
| A0A0H2UP92 | 15.75 | 2 | 254 | 25.33 | livG | SP_0752 | - | - | - | - | Cytosolic |
| A0A0H2UQS6 | 13.37 | 5 | 486 | 24.99 |  | SP_1421 | - | - | - | - | Cytosolic |
| P67506 | 26.07 | 5 | 211 | 24.79 | trmB | SP_0550 | - | - | - | - | Cytosolic |
| P66155 | 17.74 | 2 | 62 | 24.21 | rpmB | SP_0441 | - | - | - | - | Cytosolic |
| P66200 | 71.25 | 3 | 80 | 23.95 | rpmE2 | SP_1299 | - | - | - | - | Cytosolic |
| Q97PP4 | 12.89 | 4 | 450 | 23.67 | glmM | SP_1559 | - | - | - | - | Cytosolic |
| A0A0H2UQJ7 | 15.18 | 4 | 448 | 23.57 | gdhA | SP_1306 | - | - | - | - | Cytosolic |
| Q97PW5 | 16.07 | 4 | 305 | 23.51 | glyQ | SP_1475 | - | - | - | - | Cytosolic |
| A0A0H2UQH0 | 19.63 | 5 | 428 | 23.47 | hom | SP_1361 | - | - | - | - | Cytosolic |
| Q97QT2 | 12.27 | 6 | 652 | 23.37 | ligA | SP_1117 | - | - | - | - | Cytosolic |
| P0A4B3 | 33.72 | 3 | 86 | 23.26 | rpsQ | SP_0218 | - | - | - | - | Cytosolic |
| Q97NG0 | 14.92 | 4 | 449 | 23.07 | pgi | SP_2070 | - | - | - | - | Cytosolic |
| Q97P32 | 16.46 | 4 | 407 | 22.04 | trpB | SP_1812 | - | - | - | - | Cytosolic |
| A0A0H2UQ29 | 28.57 | 5 | 252 | 21.94 |  | SP_1071 | - | - | - | - | Cytosolic |
| Q97ST6 | 32.03 | 2 | 128 | 21.84 | rplQ | SP_0237 | - | - | - | - | Cytosolic |
| A0A0H2UPS3 | 8.74 | 5 | 709 | 21.38 |  | SP_0908 | - | - | - | - | Cytosolic |
| P66524 | 37.93 | 3 | 58 | 20.52 | rpsU | SP_1414 | - | - | - | - | Cytosolic |
| A0A0H2UNG6 | 14.98 | 5 | 474 | 20.22 | gnd | SP_0375 | - | - | - | - | Cytosolic |
| A0A0H2UPF3 | 76.86 | 47 | 350 | 2615.53 | pnrA | SP_0845 | - | SPII | - | - | Lipoproteins |
| P18791 | 72.99 | 48 | 659 | 1307.17 | amiA | SP_1891 | - | SPII | - | - | Lipoproteins |
| P59213 | 74.47 | 48 | 423 | 838.2 | malX | SP_2108 | - | SPII | - | - | Lipoproteins |
| A0A0H2URD1 | 77.38 | 35 | 442 | 645.41 | satA | SP_1683 | - | SPII | - | - | Lipoproteins |
| A0A0H2UPD6 | 81.35 | 26 | 386 | 599.79 | livJ | SP_0749 | - | SPII | - | - | Lipoproteins |
| Q97R51 | 74.12 | 32 | 313 | 574.61 | prsA | SP_0981 | - | SPII | - | - | Lipoproteins |
| P35592 | 75.61 | 44 | 660 | 500.22 | aliA | SP_0366 | - | SPII | - | - | Lipoproteins |
| P0A4G2 | 72.49 | 21 | 309 | 482.72 | psaA | SP_1650 | - | SPII | - | - | Lipoproteins |
| A0A0H2UPT5 | 68.62 | 21 | 341 | 442.02 | PiuA | SP_1032 | - | SPII | - | - | Lipoproteins |
| A0A0H2UQY7 | 67.63 | 31 | 278 | 434.28 | aatB | SP_1500 | - | SPII | - | - | Lipoproteins |
| A0A0H2UN58 | 78.99 | 26 | 276 | 332.96 |  | SP_0148 | - | SPII | - | - | Lipoproteins |
| A0A0H2UN92 | 75 | 27 | 284 | 313.6 | metQ | SP_0149 | - | SPII | - | - | Lipoproteins |
| A0A0H2UP74 | 60.08 | 15 | 238 | 278.06 | dacB | SP_0629 | - | SPII | - | - | Lipoproteins |
| A0A0H2URB7 | 62.92 | 29 | 445 | 269.34 |  | SP_1690 | - | SPII | - | - | Lipoproteins |
| A0A0H2UNC7 | 81.48 | 19 | 189 | 268.65 |  | SP_0191 | - | SPII | - | - | Lipoproteins |
| A0A0H2UPH6 | 67.79 | 15 | 267 | 248.33 | SlrA | SP_0771 | - | SPII | - | - | Lipoproteins |
| A0A0H2UMY0 | 60.29 | 26 | 491 | 232.33 |  | SP_0092 | - | SPII | - | - | Lipoproteins |
| A0A0H2UQR1 | 69.37 | 18 | 271 | 227.84 |  | SP_1394 | - | SPII | - | - | Lipoproteins |
| Q97Q31 | 55.82 | 13 | 292 | 202.43 | pstS1 | SP_1400 | - | SPII | - | - | Lipoproteins |
| A0A0H2US66 | 65.37 | 19 | 335 | 194.36 |  | SP_2197 | - | SPII | - | - | Lipoproteins |
| A0A0H2URK5 | 72.9 | 19 | 321 | 174.95 | piaA | SP_1872 | - | SPII | - | - | Lipoproteins |
| P0A4G0 | 41.1 | 20 | 652 | 146.87 | aliB | SP_1527 | - | SPII | - | - | Lipoproteins |
| O05703 | 50.5 | 17 | 501 | 129.61 | adcA | SP_2169 | - | SPII | - | - | Lipoproteins |
| A0A0H2URJ7 | 48.69 | 13 | 419 | 88.69 | rafE | SP_1897 | - | SPII | - | - | Lipoproteins |
| A0A0H2UPK1 | 41.38 | 9 | 290 | 67.69 |  | SP_0899 | - | SPII | - | - | Lipoproteins |
| A0A0H2UPR5 | 38.38 | 7 | 185 | 38.61 | Etrx2 | SP_1000 | - | SPII | - | - | Lipoproteins |
| A0A0H2UNA1 | 23.68 | 2 | 152 | 23.55 |  | SP_0198 | - | SPII | - | - | Lipoproteins |
| A0A0H2US70 | 75 | 27 | 392 | 729.12 | usp45 | SP_2216 | - | SPI | - | - | Secreted proteins |
| A0A0H2US63 | 62.6 | 22 | 393 | 236.55 |  | SP_2239 | - | SPI | - | - | Secreted proteins |
| A0A0H2UPV6 | 68.02 | 15 | 344 | 200.45 |  | SP_1069 | - | SPI | - | - | Secreted proteins |
| A0A0H2US14 | 60.95 | 16 | 338 | 193.02 |  | SP_1942 | - | SPI | - | - | Secreted proteins |
| A0A0H2US81 | 70.96 | 11 | 272 | 109.3 | mreC | SP_2218 | - | SPI | - | - | Secreted proteins |
| A0A0H2UPH1 | 45.52 | 10 | 413 | 90.26 | dacA | SP_0872 | - | SPI | - | - | Secreted proteins |
| A0A0H2UPZ4 | 34.57 | 10 | 324 | 76.27 |  | SP_1027 | - | SPI | - | - | Secreted proteins |
| A0A0H2UN27 | 32.63 | 4 | 95 | 63.06 |  | SP_0109 | - | SPI | - | - | Secreted proteins |
| A0A0H2XDQ8 | 27.62 | 7 | 467 | 44.4 |  | SP_1954 | - | SPI | - | - | Secreted proteins |
| A0A0H2UQS8 | 74.59 | 45 | 551 | 641.52 | mltG | SP_1518 | - | - | TM(1) | TM(1) | transmembrane |
| Q04707 | 64.12 | 38 | 719 | 564.47 | ponA | SP_0369 | - | - | TM(1) | TM(1) | transmembrane |
| A0A0H2URT5 | 55.13 | 34 | 731 | 275.05 | pbp2A | SP_2010 | - | - | TM(1) | TM(1) | transmembrane |
| A0A0H2UQ91 | 33.7 | 23 | 721 | 242.51 |  | SP_1241 | - | SPI | TM(3) | TM(3) | transmembrane |
| O69076 | 50.46 | 25 | 652 | 240.51 | ftsH | SP_0013 | - | - | TM(2) | TM(2) | transmembrane |
| Q97PA9 | 39 | 21 | 659 | 212.01 | stkP | SP_1732 | - | - | TM(1) | TM(1) | transmembrane |
| A0A0H2UNG8 | 43.89 | 12 | 303 | 202.88 |  | SP_0282 | - | - | TM(4) | TM(4) | transmembrane |
| P0A4M7 | 38.35 | 20 | 498 | 189.98 | amiC | SP_1890 | - | SPI | TM(6) | TM(6) | transmembrane |
| P14677 | 49.87 | 26 | 750 | 185.33 | pbpX | SP_0336 | - | - | TM(1) | TM(1) | transmembrane |
| A0A0H2URQ4 | 51.88 | 14 | 345 | 183.26 |  | SP_1967 | - | - | TM(1) | TM(1) | transmembrane |
| A0A0H2UPI5 | 66.17 | 16 | 399 | 180.86 |  | SP_0785 | - | - | TM(1) | TM(1) | transmembrane |
| A0A0H2UQQ3 | 50.97 | 17 | 463 | 169.74 | pgdA | SP_1479 | - | - | TM(1) | TM(1) | transmembrane |
| A0A0H2UQU8 | 28.88 | 18 | 914 | 162.85 |  | SP_1551 | - | - | TM(9) | TM(8) | transmembrane |
| A0A0H2URX1 | 32.16 | 21 | 821 | 161.61 | pbp1B | SP_2099 | - | - | TM(1) | TM(1) | transmembrane |
| A0A0H2UQH2 | 54.48 | 20 | 424 | 161.35 |  | SP_1368 | - | - | TM(2) | TM(1) | transmembrane |
| A0A0H2UP94 | 37.66 | 12 | 308 | 126.99 | ftsX | SP_0757 | - | - | TM(4) | TM(4) | transmembrane |
| A0A0H2UQG8 | 43.9 | 14 | 344 | 121.11 |  | SP_1363 | - | - | TM(2) | TM(2) | transmembrane |
| Q97SJ6 | 53.04 | 8 | 230 | 119.51 | cpsC | SP_0348 | - | - | TM(2) | TM(1) | transmembrane |
| P0A3M5 | 41.32 | 18 | 680 | 109.25 | penA | SP_1673 | - | - | TM(1) | TM(1) | transmembrane |
| Q97RK0 | 29.57 | 13 | 575 | 107.28 | ezrA | SP_0807 | - | - | TM(1) | TM(1) | transmembrane |
| P0A2Z2 | 59.76 | 10 | 164 | 105.18 | atpF | SP_1512 | - | - | TM(1) | TM(1) | transmembrane |
| A0A0H2UQR5 | 21.8 | 6 | 289 | 93.98 |  | SP_1491 | - | - | TM(6) | TM(6) | transmembrane |
| A0A0H2UQI3 | 64.52 | 12 | 186 | 89.81 | lemA | SP_1284 | - | - | TM(1) | TM(1) | transmembrane |
| P67282 | 35.58 | 16 | 534 | 83.04 | rny | SP_1739 | - | - | TM(1) | TM(1) | transmembrane |
| A0A0H2UNP9 | 32.63 | 12 | 521 | 81.72 |  | SP_0453 | C-LPxTG*^a^* | SPI | TM(3) | TM(5) | transmembrane |
| A0A0H2UMZ0 | 22.4 | 12 | 616 | 70.86 |  | SP_0103 | - | - | TM(4) | TM(4) | transmembrane |
| A0A0H2UR55 | 27.24 | 9 | 492 | 68.5 |  | SP_1715 | - | - | TM(6) | TM(6) | transmembrane |
| Q97SR2 | 35.08 | 9 | 419 | 60.03 |  | SP_0263 | - | - | TM(5) | TM(4) | transmembrane |
| A0A0H2UR65 | 15.63 | 8 | 627 | 59.73 |  | SP_1722 | - | - | TM(10) | TM(10) | transmembrane |
| Q97NP5 | 18.83 | 7 | 308 | 57.43 | yidC1 | SP_1975 | - | SPII | TM(4) | TM(4) | transmembrane |
| P35597 | 17.61 | 9 | 778 | 55.84 | exp7 | SP_1623 | - | - | TM(10) | TM(9) | transmembrane |
| P67293 | 43.9 | 3 | 82 | 55.21 |  | SP_1882 | - | - | TM(1) | TM(1) | transmembrane |
| P35595 | 14.88 | 7 | 726 | 53.64 | exp5 | SP_0758 | - | - | TM(8) | TM(9) | transmembrane |
| A0A0H2UNG3 | 22.41 | 7 | 464 | 53.36 | mapZ | SP_0374 | - | - | TM(1) | TM(1) | transmembrane |
| A0A0H2URK8 | 8.4 | 5 | 655 | 50.97 |  | SP_1884 | - | - | TM(9) | TM(10) | transmembrane |
| A0A0H2UNF1 | 28.69 | 9 | 481 | 45.33 | cps4A | SP_0346 | - | - | TM(3) | TM(3) | transmembrane |
| A0A0H2UR49 | 27.22 | 2 | 158 | 43.99 |  | SP_1604 | - | - | TM(1) | TM(1) | transmembrane |
| A0A0H2UP83 | 38.89 | 6 | 126 | 43.74 |  | SP_0678 | - | - | TM(1) | TM(1) | transmembrane |
| A0A0H2UNI5 | 28.91 | 5 | 211 | 38.64 | cps4E | SP_0350 | - | - | TM(1) | TM(1) | transmembrane |
| A0A0H2UPH7 | 17.08 | 6 | 650 | 37.17 |  | SP_0877 | - | - | TM(9) | TM(9) | transmembrane |
| P35596 | 21.38 | 9 | 608 | 37.12 | glpO | SP_2185 | - | - | - | TM(1) | transmembrane |
| P64166 | 12.91 | 7 | 767 | 36.81 | ftsK | SP_0878 | - | - | TM(5) | TM(5) | transmembrane |
| P0A3S3 | 38.69 | 6 | 274 | 36.43 | endA | SP_1964 | - | - | TM(1) | TM(1) | transmembrane |
| A0A0H2URM9 | 25.49 | 8 | 506 | 36.2 | proWX | SP_1860 | - | - | TM(7) | TM(6) | transmembrane |
| A0A0H2UR15 | 25.99 | 8 | 531 | 36.19 |  | SP_1548 | - | - | TM(2) | TM(3) | transmembrane |
| A0A0H2URJ8 | 32.91 | 4 | 158 | 36 |  | SP_1926 | - | - | TM(1) | TM(2) | transmembrane |
| A0A0H2US74 | 22.83 | 6 | 276 | 33.26 |  | SP_2223 | - | - | TM(1) | TM(1) | transmembrane |
| A0A0H2UNL7 | 22.98 | 7 | 409 | 32.72 | cps4F | SP_0351 | - | - | - | TM(1) | transmembrane |
| A0A0H2UQY6 | 22.09 | 5 | 249 | 30.29 |  | SP_1624 | - | SPI | TM(1) | TM(1) | transmembrane |
| A0A0H2USC5 | 14.36 | 6 | 564 | 30.07 |  | SP_2075 | - | - | TM(6) | TM(6) | transmembrane |
| A0A0H2UPL7 | 5.68 | 2 | 229 | 29.15 |  | SP_0858 | - | - | TM(4) | TM(4) | transmembrane |
| P0CB59 | 5.83 | 3 | 326 | 26.31 | mraY | SP_0337 | - | - | TM(8) | TM(10) | transmembrane |
| A0A0H2UQU4 | 19.92 | 4 | 256 | 25.75 |  | SP_1561 | - | - | TM(1) | TM(2) | transmembrane |
| O07344 | 15.69 | 3 | 204 | 25.56 | lepB | SP_0402 | - | - | TM(1) | TM(1) | transmembrane |
| A0A0H2URY7 | 17.69 | 5 | 605 | 23.83 |  | SP_2057 | - | - | TM(11) | TM(12) | transmembrane |
| A0A0H2UPX4 | 50.43 | 4 | 115 | 23.65 |  | SP_0990 | - | - | TM(1) | TM(1) | transmembrane |
| A0A0H2UMX9 | 14.41 | 3 | 354 | 22.73 |  | SP_0097 | - | - | TM(5) | TM(5) | transmembrane |
| A0A0H2US28 | 17.91 | 4 | 335 | 21.02 |  | SP_2132 | - | - | TM(2) | TM(2) | transmembrane |
| A0A0H2UPQ2 | 30.45 | 4 | 266 | 20.09 | lytC | SP_0987 | - | - | TM(1) | TM(1) | transmembrane |

*a* LPxTG motif was present, but the topology prediction indicates that region is present in the cytoplasmic region, so might not be available for attachment of sortase.
