# Supplementary material for: Immunomodulatory Effects of Pneumococcal Extracellular Vesicles on Cellular and Humoral Host Defenses
Source: mBio. 2018 Apr 10;9(2):e00559-18. doi: 10.1128/mBio.00559-18 (PMC5893880; doi:10.1128/mBio.00559-18)
Supplement: TABLE S2 [file mbo002183824st2.docx]

**Table S2.** **Pneumococcal virulence factors and their presence in EVs**

| **Virulence factor** | **Protein function** | **Present in EVs** | **Choline-binding domain (CBD) /LPxTG proteins** | **Topology**  **Prediction** |
| --- | --- | --- | --- | --- |
| AdcA | Zinc-binding lipoprotein | + |  | Lipoproteins |
| AliA | Oligopeptide-binding protein | + |  | Lipoproteins |
| AliB | Oligopeptide-binding protein | + |  | Lipoproteins |
| AmiA | Oligopeptide-binding protein | + |  | Lipoproteins |
| BgaA | β-galactosidase |  | C-LPxTG | Cell wall |
| CbpD | Murein hydrolase |  | CBD | Cell wall |
| CbpE | Phosphorylcholine esterase | + | CBD | Cell wall |
| CbpF | Autolysis regulator | + | CBD | Cell wall |
| CiaH | Histidine kinase |  |  | Transmembrane |
| DacB | L,D-carboxypeptidase; | + |  | Lipoproteins |
| Eno | Enolase | + |  | Cytosolic |
| Etrx1 | Thiol-disulfide oxidoreductase |  |  | Lipoproteins |
| Etrx2 | Thiol-disulfide oxidoreductase | + |  | Lipoproteins |
| IgA | IgA1 protease |  | N-LPxTG | Cell wall |
| LytA | Autolysin | + | CBD | Cell wall |
| LytB | Peptidoglycan hydrolase | + | CBD | Cell wall |
| LytC | Lysozyme | + | CBD | Cell wall |
| MetQ | Methionine-binding lipoprotein Q | + |  | Lipoproteins |
| NanA | Neuraminidase |  | C-LPxTG*^a^* | Cell wall |
| NanB | Neuraminidase |  |  | Secreted proteins |
| NanC | Neuraminidase |  |  | Secreted proteins |
| NanR | Transcriptional activator of nan operon |  |  | Cytosolic |
| PavA | Fibronectin-binding protein |  |  | Cytosolic |
| PavB | Adhesin |  | C-LPxTG | Cell wall |
| PcpA | Adhesin | + | CBD | Cell wall |
| PhtA | Zinc and manganese scavenger |  |  | Cytosolic |
| PhtB | Zinc and manganese scavenger |  |  | Lipoproteins |
| PiaA | Iron-compound ABC transporter | + |  | Lipoproteins |
| PiuA | Iron-compound ABC transporter | + |  | Lipoproteins |
| Ply | Pore-forming toxin | + |  | Cytosolic |
| PnrA | Pneumococcal nucleoside receptor A | + |  | Lipoproteins |
| PrsA | Foldase | + |  | Lipoproteins |
| PsaA | Adhesin | + |  | Lipoproteins |
| PspA | Adhesin | + | CBD | Cell wall |
| PspC | Adhesin | + | CBD | Cell wall |
| PsrP | Adhesin |  | C-LPxTG | Cell wall |
| SatA | Carbohydrate ABC transporter | + |  | Lipoproteins |
| SatB | Carbohydrate ABC transporter |  |  | Transmembrane |
| SatC | Carbohydrate ABC transporter |  |  | Transmembrane |
| SlrA | Peptidyl-prolyl cis-trans isomerase | + |  | Lipoproteins |
| SpxB | Pyruvate oxidase | + |  | Cytosolic |
| StrH | β-D-N-acetyl-hexosaminidase |  | N-LPxTG | Cell wall |

*a* Pneumococcus nanA has a C-terminal LPxTG cell wall anchoring domain, however nanA is truncated in Tigr4 strain and may not possess LPxTG domain.
