# Supplementary material for: Immunomodulatory Effects of Pneumococcal Extracellular Vesicles on Cellular and Humoral Host Defenses
Source: mBio. 2018 Apr 10;9(2):e00559-18. doi: 10.1128/mBio.00559-18 (PMC5893880; doi:10.1128/mBio.00559-18)
Supplement: TABLE S3 [file mbo002183824st3.docx]

**Table S3. Human serum proteins bound by pneumococcal EVs**

| **Accession** | **Protein name** | **Score** | **MW (kDa)** |
| --- | --- | --- | --- |
| P04114 | Apolipoprotein B-100 | 837.85 | 515.3 |
| P08519 | Apolipoprotein(a) | 21.89 | 501.0 |
| P01024 | Complement C3 | 300.60 | 187.0 |
| P01031 | Complement C5 | 205.10 | 188.2 |
| A0A0G2JL54 | Complement C4-B | 75.14 | 187.6 |
| A0A087X130 | Ig kappa chain C region | 33.85 | 25.1 |
| P02747 | Complement C1q subcomponent subunit | 25.63 | 25.8 |

| **Accession** | **Protein name** | **Score** | **MW (kDa)** |
| --- | --- | --- | --- |
| P04114 | Apolipoprotein B-100 | 837.85 | 515.3 |
| P08519 | Apolipoprotein(a) | 21.89 | 501.0 |
| P01024 | Complement C3 | 300.60 | 187.0 |
| P01031 | Complement C5 | 205.10 | 188.2 |
| A0A0G2JL54 | Complement C4-B | 75.14 | 187.6 |
| A0A087X130 | Ig kappa chain C region | 33.85 | 25.1 |
| P02747 | Complement C1q subcomponent subunit | 25.63 | 25.8 |
